# Application of a uniaxial force by pulling the skin around the mammary gland may affect the orientation of the ducts and the length of the mammary ductal network: Findings from computational modeling and laboratory experiments

**DOI:** 10.1371/journal.pcbi.1014421

**Published:** 2026-07-14

**Authors:** Daisy Ulloa, Kelsey M. Teeple, Sara B. Scinto, Wonders O. Ogundare, Deloris D. Franklin, Theresa M. Casey, Uduak Z. George

**Affiliations:** 1 Department of Mathematics and Statistics, San Diego State University, San Diego, California, United States of America; 2 Computational Science Research Center, San Diego State University, San Diego, California, United States of America; 3 Department of Animal Sciences, Purdue University, West Lafayette, Indiana, United States of America; University of California Riverside, UNITED STATES OF AMERICA

## Abstract

The orientation of the epithelial ducts determines the overall shape of the ductal network in the mammary gland. However, how the orientation of the duct is specified is not well understood. This study examined if an externally applied tensional force alters the orientation of the epithelial ducts during puberty, *in vivo*. In the first experiment, uniaxial forces were applied continuously to mouse mammary glands from 5-7 weeks of age, that is, during pubertal formation of the epithelial ductal network. The uniaxial forces were applied by pulling and adhering the skin around the nipple of the right abdominal number four mammary glands (n = 10; TENSION). For experiment two, the uniaxial force was applied from 5-6 weeks of age, and glands from half the mice (n = 10/group; TENSION and control (CTL)) were collected. In the remaining mice, the application of force was removed at 6 weeks from the TENSION group, and then the glands were collected at 7 weeks of age (n = 10/group; TENSION and CTL). This pulling force on the right gland made the left abdominal number four gland also experience a contralateral (CONTRA) force. Following dissection, whole mounts were prepared, and panoramic images were captured. The ductal network in the images was skeletonized and straightened along the longitudinal midline to capture the length of the ductal network without bias from its curvature. Findings indicated that there was no difference in ductal area in either experiment, but the length of the ductal network was increased in the TENSION and CONTRA mammary glands compared to CTL (p < 0.05) when uniaxial force was applied from 5-7 weeks. Moreover, the orientation of the ducts in the TENSION and CONTRA mammary glands was altered compared to CTL (p < 0.05) in both experiments. In-silico simulations of the ductal network formation using a branching and annihilating random walk model predicted that the increased length of the ductal network may have resulted from changes in the orientation of the epithelial ducts. Thus, it is likely that mechanical forces regulate the orientation of ductal branches *in vivo*.

## Author summary

Although the formation of the epithelial ductal network in the female mouse mammary gland begins at the embryonic stage, it remains rudimentary until puberty. During puberty, a complex network of ductal branches forms from the rudimentary ducts. Some processes involved in the formation of the ductal network are dysregulated in diseases such as breast cancer; therefore, research on ductal network formation is vital for understanding how things break down in disease. Cell-cell and cell-extracellular matrix interactions perturb the tissue mechanical environment, leading to cues that regulate processes such as the morphogenesis of the ductal network. However, how the orientation of the epithelial ducts in the ductal network is specified is not well understood. Therefore, in this study, we applied a uniaxial force by adhering the skin around the nipple of the mammary gland to investigate how an externally applied mechanical force may affect the organization of the ducts in the mammary gland during pubertal development. Results from the experiment, in combination with simulations, indicate that the length of the ductal network and the orientation of the ducts were altered in the experimental group compared to controls. Thus, it is likely that mechanical forces regulate the orientation of ductal branches *in vivo*.

## 1 Introduction

Tissue mechanical environment plays a major role in the proper formation of epithelial and glandular tissues with tree-like structures, such as the lung airways, the salivary gland, the pancreatic ducts, and the mammary epithelial ducts [1]. These tree-like structures are critical for the long-term physiological functioning of organs, and developmental defects often lead to poor health outcomes [2–6]. Branching morphogenesis is the developmental process that governs the formation of these tree-like structures. A great portion of the formation of the lung airways, the salivary gland, and the pancreatic ductal network occurs during the embryonic and fetal stages of development. Although the formation of the mouse mammary ductal network begins during the embryonic stage of development, it remains rudimentary and composed of a primary duct and 15–20 ductal branches until puberty [7]. The female mammary gland is a great model for studying branching morphogenesis because the expansion from a rudimentary structure to a complex epithelial ductal network occurs postnatally during puberty, allowing for investigations into the evolution of these complex network structures after birth, *in vivo*. The expansion of the mammary ductal network begins at about 4 weeks old in mice, under the influence of hormones. At 10 weeks, the mammary gland is largely infiltrated by a complex network of ducts, which will undergo cycles of branching and regression throughout each estrous cycle [7].

Many factors are known to regulate branching morphogenesis in the mammary gland. Upon examining the morphogenesis of mammary epithelial tubules of varying geometry in culture, Nelson et al. [8] found that the position where branching initiates depends on the initial geometry of the tubule. They also found that the sites where branching initiates correspond to the positions where the concentration of self-secreted inhibitory signals, such as transforming growth factor β, is locally minimized in the tubules, thereby implicating the geometry of the tubule in dictating the site where branching occurs. Interactions between epithelial cells and their surrounding stromal microenvironment instruct branching morphogenesis, but the underlying mechanisms remain incompletely understood [9]. Using a mammary organoid system, built by coculturing epithelial cells, fibroblasts, and endothelial cells, Wang et al. [10] found that fibroblasts and endothelial cells grow along with the epithelium, clustering within the organoid core. They further demonstrated that both cell types are essential in generating branching organoids, and that ablation of either fibroblasts or endothelial cells resulted in a significant reduction in the frequency of organoid branches. Sumbal et al. [9] showed that in a mammary organoid co-culture of mammary epithelial organoids with mammary fibroblasts, physical contact between fibroblasts and epithelial cells and fibroblast contractility are required to induce mammary epithelial branching, identifying mechanical activity as a critical regulator of epithelial morphogenesis.

How the orientation of the epithelial ductal branches is specified remains an open question [1,11–13]. A crucial step for elucidating how the orientation of the epithelial ductal branches is specified is to identify the mechanisms that modulate or regulate the branch orientation *in vivo*. This would facilitate the selection of potential regulatory candidates and enable the formulation of hypotheses that can be tested through laboratory experiments and computational modeling. Although it is known that mechanical forces affect branching morphogenesis, their effect is not fully understood nor well characterized in the mammary gland. Therefore, in this study, we perturbed the mechanical environment by application of an external uniaxial force and determined if and how it modifies the orientation of the epithelial ducts in the mammary gland, *in vivo*.

**Fig 1:**
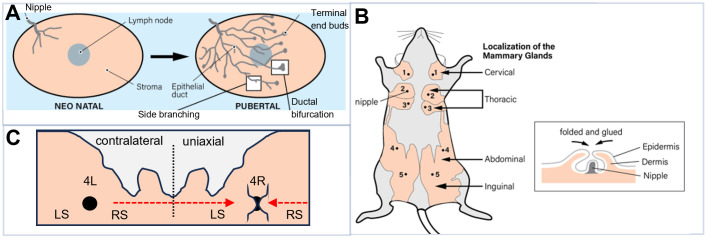
Overview of pubertal branching morphogenesis and laboratory experimentation. (A) Neonatal versus pubertal mammary gland. The formation of an extensive network of epithelial ducts occurs during pubertal development via ductal elongation, tip bifurcation, and side branching. Figure modified from Wiseman and Werb [14]. (B) The right abdominal number four mammary gland was used to investigate the role of tensional force on mouse branching morphogenesis. Figure of mouse modified from Honvo-Houéto and Truchet [15]. The inset shows how a uniaxial tension force was applied to the right abdominal number four mammary gland. The skin surrounding the nipple was lifted, folded, and glued together as shown.(C) A schematic showing the direction of forces applied for uniaxial (TENSION) and contralateral (CONTRA) glands. Here, TENSION and CONTRA glands correspond with the right(4R) and left (4L) number 4 nipple, respectively. LS and RS denote the left and right sides of the gland, respectively. The red arrows indicate the direction of forces applied. TENSION glands experience direct force on both 4R LS and 4R RS, whereas CONTRA glands experience indirect force on the 4L RS towards the 4R LS.

During puberty development of the mammary gland, an extensive network of epithelial ducts emerges from a rudimentary ductal structure via the process of branching morphogenesis (Fig 1A) [16,17]. In the mammary gland, branching morphogenesis occurs via two main forms [14]: the first is through successive rounds of elongation and splitting of the tips of existing parent ducts, known as tip bifurcation. The second form of branching is through the elongation of buds that protrude along the sides of existing ducts, known as side branching [18]. A majority of the studies on mammary ductal network formation have focused on identifying the molecular factors that regulate its development, including ductal elongation, tip bifurcation, and side branching [17,19–27]. Comparatively fewer studies aimed to elucidate the role of mechanical forces in the formation of the mammary ductal network.

In two-dimensional (2D) epithelial cell sheets, traction forces exerted by cells reorient and realign collagen fibers in their preferred direction of migration or tension, facilitating cell movement. It has been suggested, though not demonstrated experimentally, that this may also occur in human mammary organoids cultured in a 3D collagen gel [28]. The reorganization of collagen fibers can alter tissue stiffness and, in turn, affect cell behavior. Exogenous forces, such as an applied uniaxial force, are also capable of modifying the organization of the collagen [29], which would affect tissue stiffness. Cells can sense stiffness and respond through the process of mechanotransduction. Mechanical signaling can lead to functional and structural responses in the cells and tissues by influencing cell adhesion, migration, morphology, and differentiation [30,31], which are key processes involved in branching morphogenesis [32].

Observations of branching morphogenesis in organoid models demonstrate that the mechanical properties of the region around the ductal branch can direct branch elongation [28] and bias ductal growth along the long axis of the developing gland [33]. Though organoids provide excellent models for studying branching morphogenesis, they are not able to model the complexity of the mammary branched structure and the *in vivo* mammary gland environment [34,35]; therefore, *in vivo* studies are needed to further our understanding of the role of mechanical forces in the formation of the ductal network. In this study, we aimed to determine whether mechanical forces, applied *in vivo*, can regulate the orientation of the epithelial ductal branches. In order to determine whether mechanical forces can regulate the orientation of the ductal branches, a uniaxial force was applied to the right abdominal number four mammary gland in mice for two weeks during pubertal development (Fig 1B, inset). Thereby altering the mechanical environment of the entire gland (Fig 1A) [36–38].

Microscopy images of mouse mammary glands subjected to uniaxial force in situ during peripubertal ductal morphogenesis were analyzed and compared to unmanipulated glands to evaluate the effect of the uniaxial force on the ductal network formation. Uniaxial force applied to the right abdominal number four mammary gland induced a contralateral force on the left abdominal number four mammary gland, resulting in unilateral pulling of the left gland, whereas the right gland experienced bilaterally directed pulling. The morphology of the ductal network, including the orientation of ductal branches, the length and width of the overall ductal network, the cross-sectional area of the epithelium network, the average curvature of the ductal network, and the number of perimetral branches were examined in the glands that experience uniaxial and contralateral forces and were compared to controls. Significant differences occurred in the orientation of the ductal branches and the length of the ductal network in the glands exposed to uniaxial and contralateral forces, compared to controls. Furthermore, a branching and annihilating random walk model was applied to study why the ductal network in the mammary glands exposed to uniaxial and contralateral forces was longer than that in controls. Findings from the branching and annihilating random walk simulations indicate that a uniaxial force may regulate the length of the ductal network by modifying the orientation of the ductal branches. This study contributes to increasing our understanding of the effect of mechanical forces on the formation of the epithelial ductal networks.

## 2 Materials and methods

### 2.1 Ethics Statement

This study was carried out in strict accordance with the recommendations in the Guide for the Care and Use of Laboratory Animals of the National Institutes of Health. The protocol was approved by Purdue Univeristy’s Institutional Animal Care and Use Committee (IACUC no. 190100845 and 0123002338), with the gradual-fill method of carbon dioxide inhalant euthanasia of mature mice applied in these studies consistent with approved approaches by the American Veterinary Medical Association [39].

### 2.2 Laboratory experimentation: Application of the uniaxial tension force to the mammary glands

A total of 70 mice were used for these experiments. Mice were group housed by treatment, with 5 animals per cage. During the entire study, mice were given ad libitum access to water and standard mouse chow, and were exposed to 12 h light and 12 h dark cycles.

Two experiments were conducted. For the first experiment, referred to as the primary experiment, mice (n = 32) used were offspring born to Wap-cre (B6129-Tg(Wap-cre)11738Mam/J) and wild type C57BL/6J crosses (Jackson Laboratory, Bar Harbor, ME) bred in the Casey laboratory at Purdue University. Genotypes of offspring produced by this cross are either wild type or heterozygous Wap-cre. Heterozygous Wap-cre offspring do not show an overt mammary phenotype, and so although mice were genotyped, both wild-type and heterozygous female offspring carrying the Wap-cre allele were used for the studies. The mice used were the F2 generation of these crosses. Parent generation (P0) received from Jackson Laboratory was generated by in vitro fertilization of the C3H strain of mice with cryopreserved sperm of B6129-Tg(Wap-cre)11738Mam/J mice. Choice of the C3H strain for cryorecovery is due to its high fertility and fecundity. To ensure the stage of the progression of mammary development was consistent with the literature of C57BL/6J, glands were collected from mice at 3 weeks of age (n = 6) and at 5 weeks of age (n = 6), and whole mounts were prepared. Visual inspection confirmed mammary glands were normally developed, and the stage of development was consistent with the age of the mouse.

The remaining female offspring (n = 20) were randomly assigned to one of two groups: control (CTL, n = 10) and experimental group (n = 10). At five weeks of age, the female mice were anesthetized using 3% isoflurane gas with a flow rate of 1.0 L/minute oxygen. The fur surrounding the nipple of the right abdominal number four mammary gland was removed by plucking with forceps from all mice (both CTL and experimental groups). The skin on either side of the right nipple was lifted and glued together using topical tissue adhesive (GLUture, Zoetis Inc., Kalamazoo, MI, USA) to encase the nipple (Figs 1B and S1), exerting a uniaxial force on the mammary gland. The aim was to apply similar tension across animals, and so the skin was folded and affixed with a similar angle to midline across mice. These glands were referred to as TENSION glands (n = 10); the left abdominal number four mammary gland was referred to as the contralateral gland (CONTRA; n = 10) (Fig 1C). As an analogy for reader understanding, the skin can be envisioned as a secured, fitted sheet on a bed. If the sheet is pulled up and over and secured with a clothespin (or glue) to remove looseness on one half of the bed, it will affect the tension of the sheet on the other half of the bed. The glue was checked daily. If the glue was removed, the mice were anesthetized, and the skin was lifted and glued again. At 7 weeks of age, the mice in the experimental and control groups were euthanized, and whole mounts of mammary glands were prepared for analysis.

Mice (n = 40) for the second experiment were wild-type C57BL/6J obtained from Jackson Laboratory at 3 weeks of age. The second experiment was designed to examine how removal of the applied force after one week affects ductal network formation. Mice were assigned to treatments at 3 weeks of age by matching by weight (n = 20/group; CTL and TENSION/CONTRA). At 5 weeks of age, glue was applied to secure the skin around the nipple of the abdominal mammary gland of the TENSION/CONTRA mice. At 6 weeks of age, half the CTL (n = 10) and half the TENSION/CONTRA (n = 10) mice were euthanized, and mammary glands were collected and prepared as whole mounts. The remaining mice at 6 weeks in the TENSION/CONTRA group (n = 10) had glue removed with gentle wiping with a damp Kimwipe to release tension. These mice and remaining controls (n = 10) were euthanized at 7 weeks of age and mammary glands collected for whole mount preparation. The second experiment is hereafter referred to as the secondary experiment.

### 2.3 Collection of the mammary glands and whole mount carmine alum staining

Immediately after euthanasia, the right abdominal number four mammary glands were removed in both the CTL and TENSION groups. Although the left abdominal four mammary glands in the TENSION groups were not directly exposed to tensional forces, the gluing procedure pulls laterally on the skin beside its nipple, thus indirectly exposing these glands to mechanical stress via contralateral forces. Therefore, the left abdominal four mammary glands were also extracted from the experimental mice for analysis and quantification of the effect of the contralateral forces on mammary gland branching morphogenesis. The extracted glands were placed on a plain frosted microscope slide (Fisher Scientific, Hampton, NJ, USA) and gently spread out with the blunt end of the forceps. After allowing the mammary whole mount to dry and adhere to the microscope slide for about 5 minutes, the slides were placed in Carnoy’s solution (75% absolute ethanol and 25% glacial acetic acid) to fix overnight. The next day, the slides were transferred into carmine alum (Stemcell Technologies Inc., Vancouver, British Columbia, CA) and stained overnight at room temperature. The following day, slides were moved into destaining solution (70% absolute ethanol, 5.64% 37% HCl, and double-distilled water) for 2 h. Next, slides were dehydrated in increasing concentrations of ethanol (70%, 80%, 95%, and 100%) for 30 minutes at a time. Lastly, slides were defatted in toluene for 30 min or until the fat was sufficiently cleared from the glands. A cover slip was applied using DPX mounting media (Sigma-Aldrich, Burlington, MA, USA) and dried overnight. Once the whole mount carmine alum slides finished drying, the slides were viewed with a M60 stereo microscope with a camera port (Leica Microsystems, Wetzlar, Germany). Images from the microscope were captured using a C-mount AmScope camera and saved using the AmScope software (AmScope, Irvine, CA, USA). Multiple images were taken to capture the entire gland by moving the slide across the span of the tissue. To create a single panorama image of the entire gland, Adobe Photoshop (V 23.0.1) was used to merge the individual images into a single image. The microscopy images of the entire mammary gland were used for morphometric analysis for CTL, TENSION, and CONTRA glands.

Although the mouse mammary gland is a three-dimensional (3D) structure, its depth is modest relative to its length and width. We note that flattening a 3D structure onto a histology slide during whole mount preparation may introduce deformation, and we recognize this as a limitation of our approach. Nevertheless, studies in the literature on mouse mammary gland morphometry are routinely performed on planar images, as they provide adequate information without the additional cost of volumetric imaging. Moreover, except for ductal area, the key morphometrics used in this study, ductal branch angles and network length, are not expected to change substantially when computed in 2D versus 3D. Branching angle measurements should therefore be interpreted as reflecting the projected planar geometry of the gland rather than its native three-dimensional architecture.

### 2.4 Morphometric analysis of the ductal network in TENSION, CONTRA and CTL glands

**Fig 2:**
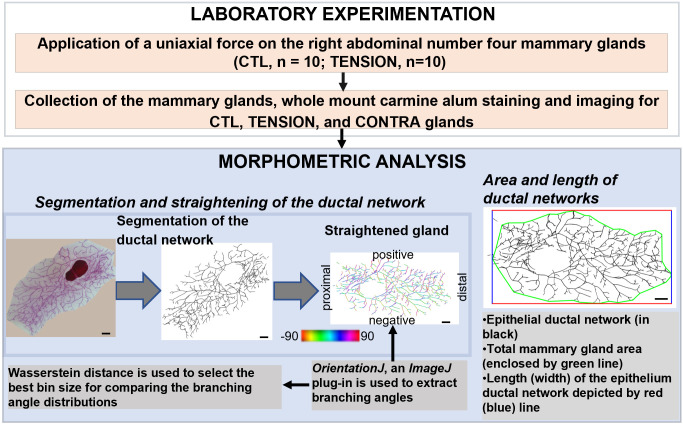
Laboratory experimentation and morphometric analysis. The color map shows high and low branching angles, measured in degrees. Morphometric measurements were obtained for all CTL, TENSION, and CONTRA glands. Terminology for orientation is also indicated as proximal - distal along the horizontal midline of the gland and positive - negative in the vertical direction. The positive and negative sides also correspond with color map branching angles. Values between 0°-90° grow towards the positive side and values between -90°-0° grow towards the negative side. Scale bar = 1 mm.

Morphometrics such as the area, length, and average curvature of ductal networks and the orientation of the epithelial ducts in the ductal network were computed (Fig 2).

*Average curvature of the ductal network:* The images of the entire mammary gland were used to compute the average of the ductal network in CTL, TENSION and CONTRA glands. This was done using the Kappa Plugin in the Fiji open-source image processing package [40,41]. The average curvature was computed as follows: points were placed on the mammary gland images along the perimeter of the ductal network and using the Kappa Plugin, the average curvature was computed by averaging the curvature of each point (S3 Fig).

*Area and length of the ductal network:* The total cross-sectional area of the ductal network for each gland was obtained using ImageJ version 1.54g [42]. First, the microscopy images were segmented using a neural network (S2A and S2B Fig) [43,44]. After, noise was removed and connected/disconnected branches were corrected by hand using Adobe Photoshop (S2C Fig). After this was completed, the total pixel in the segmented epithelial ductal network was computed by using the histogram tool in ImageJ to determine the total epithelial area. For the total cross-sectional area of the gland spanned by the ductal network, the area of a polygon that circumscribes the ductal network by closely tracing the perimeter was calculated (Fig 2 and S3). Lengths of the gland from the nipple, beginning of lymph node (defined as the side closest to nipple), and end of lymph node (defined as side farthest from nipple) to the distal tip were taken using the Measure tool in ImageJ (Fig 2 and S4-S5). To accurately compare the length of ductal networks without bias from their average curvature, the branched networks were computationally straightened to remove the curvature and ensure that the long axis of the branched network was oriented along the x-axis [33]. To straighten the branched network, we used the straighten tool in the edit menu in ImageJ. After the branched network was straightened, a bounding box starting from the nipple, beginning of lymph node, and end of lymph node to the distal tip was used to measure the length and width of the network (S5 Fig).

*Orientation of the mammary ducts:* The orientation of the mammary ducts was computed using OrientationJ version 16.01.2018 [45–47], an ImageJ plugin. To compute the orientations of the mammary ducts, the straightened ductal network was adjusted such that the proximal, distal, positive, and negative sides of the ductal network were oriented in the same direction. Maintaining consistency was important for establishing a common reference for computing and comparing ductal orientations between TENSION, CONTRA, and CTL glands. Finally, after adjusting the ductal networks to point in the same direction, the orientation of the mammary ducts in the images was computed. This was carried out using the Analysis and Distribution tool in OrientationJ.

In the Analysis and Distribution tool, the variable local window σ was set to 10 pixels when computing the local orientations of the mammary ducts. The choice of the value of σ can significantly affect the resolution of the orientation analysis as it specifies the size of the neighborhood around each pixel that is considered when computing the ductal orientation. σ was set to 10 pixels based on work from studies in the literature that demonstrate that this value of σ is adequate [45–47]. In OrientationJ, the minimum coherency and energy level was set to 0.1%, meaning pixels were detected if their levels were at or above this percent value. The angle distribution obtained from OrientationJ provided information on the number of pixels that were oriented in each integer degree value between $\left[-90^{\circ },90^{\circ }\right]$ (Fig 2). Angles above $0^{\circ }$ indicate branch growth towards the positive side of the oriented gland whereas angles below $0^{\circ }$ indicate growth towards the negative side of the oriented gland. Branching orientation was calculated relative to a fixed 0° axis rather than relative to individual branches. This approach ensures that overlapping branches are assigned distinct orientations, as each is computed independently of adjacent or crossing structures. Therefore, the presence of overlapping ducts does not compromise the accuracy of individual orientation measurements. To find the proportion of branching that lie within a certain degree range, the sum of pixels in the chosen degree range was divided by the total number of pixels measured in OrientationJ. The proportion of ducts in each degree angle was used as a measure for the orientation of the ducts in the TENSION, CONTRA, and CTL glands. The distribution of the proportion of ducts in each angle range was compared between TENSION, CONTRA, and CTL glands. Wasserstein distance, a measure from the optimal transport method, was used to determine the degree of similarity in ductal orientation between TENSION, CONTRA, and CTL glands. Wasserstein distance was also used to choose the most suitable number of histogram bins for nuanced similarity comparisons.

For statistical analysis, a Mann-Whitney test was used as it is ideal for small datasets that are not normally distributed. All analysis was confirmed by an individual who was blinded to the study. For morphometric analysis, an intraclass correlation coefficient was computed to verify that measurements by both individuals did not vary. Morphometrics were averaged by both individuals and combined into a single result. The exception was the curvature measure since this is the only measure that involves the original, unaltered gland. This value was calculated by one individual tracing the perimeter of each gland. Statistical outliers were identified using the interquartile range (IQR) method, in which values falling below Q1 - 1.5× IQR or above Q3 + 1.5×IQR were flagged and removed prior to analysis. Because such values may reflect biological variation rather than technical artifacts, statistical analyses were also performed on the complete datasets including outliers, and these results are reported in Tables A - D in S1 Text for comparison.

### 2.5 Optimal transport method for computing similarities in the distribution of ductal orientations between TENSION, CONTRA, and CTL glands

Here, we introduce a novel approach that utilizes the optimal transport method to quantify the similarity in the distribution of ductal branch orientations between mammary ductal networks. Optimal transport theory provides a measure known as the Wasserstein distance W(μ,η) for computing distances between pairs of probability distributions, say μ and η. The Wasserstein distance offers several advantages in this context. First, it captures differences in the full shape of the distribution, including shifts in location, changes in spread, and multimodality, rather than relying on summary statistics such as the mean. This is particularly important because branching angle distributions in mammary gland morphogenesis are not necessarily unimodal or symmetric, making mean-based comparisons potentially insufficient. Second, we use the Wasserstein distance as an objective, data-driven criterion for selecting histogram bin size. Rather than iterating over bin sizes to obtain statistically significant results, which can introduce researcher degrees of freedom, we select the binning that maximizes the separation between control and experimental distributions as measured by the Wasserstein distance. This reduces arbitrariness and ensures that the chosen representation best reflects differences between TENSION, CONTRA, and CTL glands. Importantly, the Wasserstein distance is not used as a replacement for hypothesis testing, but as a complementary tool for optimizing data representation. Formal statistical comparisons are subsequently applied to the resulting distributions.

The Wasserstein distance W(μ,η) satisfies conditions that are desirable when comparing the similarity between two probability distributions, μ and η. One of the desirable conditions is the symmetry condition W(μ,η)=W(η,μ) [48]. The symmetry condition is desirable because it ensures that W(μ,η)=W(η,μ). Without this property, the Wasserstein distance would not qualify as a true distance metric, and we could not interpret it as a symmetric distance between distributions. Another condition that W(μ,η) satisfies is W(μ,η)=0 if and only if μ=η, which ensures that identical distributions have zero distance. Another commonly used measure for comparing probability distributions is the Kullback–Leibler (KL) divergence. The KL divergence $D_{KL}(\mu ||\eta )$ calculates a score that measures the divergence of the probability distribution μ from η defined on the same space ξ. If μ and η are discrete probability distributions, then $D_{KL}(\mu ||\eta )=\sum_{x\in \xi }\left(\mu (x)\log\left(\frac{\mu (x)}{\eta (x)}\right)\right)$ [49]. A drawback of the KL divergence is that it does not satisfy the symmetry condition, that is, $D_{KL}(\mu ||\eta )\neq D_{KL}(\eta ||\mu )$. That is, the divergence from distribution μ to η is not equal to the divergence from η to μ. Therefore, it is not a good approach for comparing similarities between our probability distributions and is often used as a measure of discrepancy between two distributions rather than a metric [49]. When measuring the similarities between the probability distributions for the branching angles between TENSION vs. CTL and CONTRA vs. CTL, the Wasserstein distance was applied because it satisfies the symmetry condition.

Let μ represent the probability distribution of the branching angles for the TENSION glands and let η represent the probability distribution of branching angles for the CTL glands. The probability distribution μ (or η) is computed by calculating the proportion of branching angles of the TENSION (or CTL) glands that lie within each angle range. For each distribution, the normalized histogram heights sum to one. Thus, we let $p_{i}$ denote the normalized height, or probability mass, assigned to source bin *i* in μ, and let $q_{j}$ denote the normalized height, or probability mass, assigned to target bin *j* in η. Therefore, $𝐩=(p_{1},p_{2},\ldots ,p_{n}{)}^{⊤}$ and $𝐪=(q_{1},q_{2},\ldots ,q_{n}{)}^{⊤}$ are vectors satisfying $\sum_{i=1}^{n}p_{i}=1$ and $\sum_{j=1}^{n}q_{j}=1$. The Wasserstein distance W(μ,η) computes the optimal (i.e., minimum) cost of moving the probability mass of μ to obtain η. The number of bins in both μ and η is denoted by *n*. The ordered bin-index location of bin *i* in μ is denoted by $x_{i}$, and the ordered bin-index locations for all the bins in μ are denoted by $\{x_{i}{\}}_{i=1}^{n}$. Similarly, the ordered bin-index locations of the bins in η are denoted by $\{x_{j}{\}}_{j=1}^{n}$. The cost $C_{ij}$ for moving one unit of probability mass from source bin *i* to target bin *j* is measured by the Euclidean distance between these ordered bin-index locations, $C_{ij}=|x_{i}-x_{j}|$, where $x_{i}$ and $x_{j}$ denote the ordered bin-index locations, and $C=(C_{ij}{)}_{1\leq i,j\leq n}$ is a symmetric matrix in ${\mathbb{R}}^{n\times n}$. A transportation plan $T\in {\mathbb{R}}^{n\times n}$ describes how much probability mass is transported from each source bin *i* to each target bin *j*. The total cost of a given transport plan is


$$\begin{array}{c} \langle T,C\rangle =\sum_{i=1}^{n}\sum_{j=1}^{n}T_{ij}C_{ij}. \end{array}$$

The optimal transport plan $T^{*}$ is obtained by solving the following optimization problem:


$$\begin{array}{c} \underset{𝐓}{\text{minimize}}\;\langle 𝐓,𝐂\rangle \end{array}$$ (1)

$$\begin{array}{c} \text{subject to}\;𝐓1=𝐩,\;{𝐓}^{⊤}1=𝐪,\;𝐓\geq 0, \end{array}$$ (2)

where 1 denotes a vector of ones. In this study, the Wasserstein distance refers specifically to the discrete 1-Wasserstein distance, denoted by ${𝒲}_{1}(\mu ,\eta )$, given by


$$\begin{array}{c} 𝒲(\mu ,\eta ):={𝒲}_{1}(\mu ,\eta )=\langle {𝐓}^{*},𝐂\rangle . \end{array}$$

This distance is used to measure the degree of similarity in branching angle distributions between TENSION versus CTL glands and CONTRA versus CTL glands. To measure the degree of similarity, the Wasserstein distance between TENSION and CTL glands is computed across multiple bin sizes, and the bin size that best differentiates the two groups is selected. The same procedure is applied to CONTRA and CTL glands.

### 2.6 Modeling ductal network orientation with a biased branching and annihilating random walk model

The biased and annihilating random walk model was used to simulate ductal network formation and study how changes in the branch angles affect the morphology of the ductal network. The simplest random walk moves forward in space, with each step taken in a randomly chosen direction. Random walk models can be described by two attributes: correlated/uncorrelated and biased/unbiased. Correlation refers to whether the direction of each step is influenced by the previous step; bias refers to whether each step has rules that influence direction, while still incorporating randomness in each step [50]. Random walk models can be used to describe movement, such as Brownian motion, as well as cellular and molecular motion [50]. Hannezo et al. [51] proposed that correlated/persistent random walks can be used to simulate the branched structure of the ductal network. They used simple rules of branching (i.e., replication) and annihilation (i.e., branch death) to create branching structures similar to those observed in mammary gland experiments. Their model involved three basic rules that defined the dynamics of the tip of the ductal branches: (i) ducts grow outward from active tips, advancing in random directions with a speed *v*, (ii) at any point in time, active tips may randomly split into two branches with a constant probability, (iii) growth ceases for an active tip when the active tip approaches an existing duct within a defined annihilation distance, causing it to deactivate. Visual inspection of their model simulations of the ductal network revealed good qualitative agreement in spatial organization with experiments [51].

Hannezo et al. [51] created an additional random walk that introduced bias toward the distal direction; however, the introduction of bias produced ductal networks that were not in good agreement with experimental data. In a follow-up study, Nerger et al. [33] conducted branching and annihilating random walk simulations that controlled for the branching angle between two bifurcating branches. Their simulation results showed that a controlled bifurcation angle is sufficient to reproduce the global bias in the epithelium observed *in vivo*. Here, bias is introduced to tend branching towards the specific angles that we observed to differentiate TENSION, CONTRA, and CTL in the laboratory experiments. The goal is to test whether a bias in branching angle alters the length of the ductal network. The rules for the Hannezo et al. model include a constant probability for bifurcation and death. Our model excluded the rule for death. Instead, death would occur if two branches intersected. Similar to Nerger et al. [33], we controlled for the bifurcation angle, but at a broader range. Nerger et al. controlled for bifurcation angles of approximately 75°, whereas we allowed for bifurcation angles from 30° - 90° to allow for bias in the movement.

As stated, bias was introduced into the random walk model at each step by limiting the randomness of the direction to move towards a given angle. To introduce bias, a function referred to as Biased Random Value was used. This function works by picking an integer from a window centered about the current angle $\alpha_{0}$, and assigning weights to each integer in the window. The window ranges from [$\alpha_{0}$ + 15, $\alpha_{0}$ - 15] in degrees, where each value in the window is divisible by 5. Values with a higher weight are more likely to be chosen, whereas lower weights have less chance of being chosen. Choices from the Random package in Python version 3.10.9 was used to randomly select a value from the list. Choices was used because it can apply weights to a list of numbers and then randomly select a number from the list while also taking the weight of each number into consideration. The biased random value function is described as follows.

*Biased Random Value function:* (i) Set the values for the current branching angle $\alpha_{0}$ and the bias *b*_0_. (ii) If say $\alpha_{0}=10^{\circ }$ and $b_{0}=20^{\circ },$ then [$\alpha_{0}$ + 15, $\alpha_{0}$ - 15]:= [25,-5] degrees and the window vector, denoting the possible branching angles that the next step in the random walk model can take: is [25, 20, 15, 10, 5, 0, -5] degrees, (iii) reorder values from smallest to largest absolute distance from the bias to obtain [20, 25, 15, 10, 5, 0, -5] degrees, (iv) create list of weights in descending order: [20%, 17%, 17%, 13%, 13%, 10%, 10%] where reordered window list at position *i*, is associated with weight at position *i*, (v) use Choices from the Random package with the reordered list and weights as input to choose a new angle value θ as the branching angle. Values associated with a higher weight are more likely to be randomly chosen from the list. In this example, angle 20 is more likely to be chosen since the bias is 20.

**Fig 3:**
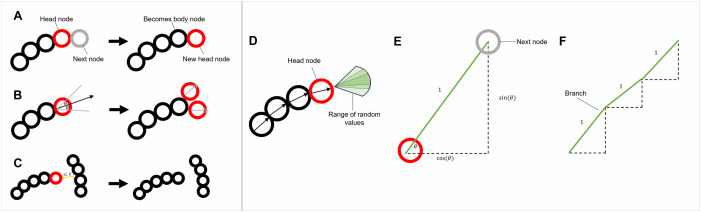
A Biased branching and annihilating random walk model. (A) Shows how the head node property changes to a body node as a branch elongates or bifurcates. (B) shows how bifurcation occurs in the algorithm. (C) shows how the intersection functions detect other branches. (D) shows how the head node will choose the angle for the next node. (E) shows the placement of the next node based on the angle chosen in (D). (F) shows how the branch will form over several elongations.

Our model includes a set of rules for elongation, bifurcation, and intersection events. Each of these rules use a branch head node as input. The head node is defined as the most recent node of an alive branch from which the events elongation, bifurcation, or death can occur. Once an event has concluded, the head node will become a body node that is dormant (Fig 3A). The rules also depend on information from a matrix that holds the (*x*,*y*) coordinates of all body and head nodes. This matrix is referred to as Tracker. The Intersection algorithms (Fig 3C) are a series of true/false algorithms that check for intersections between the new head node and existing branches or the boundary during elongation. If the new head node intersects another branch, the current head node will die and not elongate to the new head node. Otherwise, the current head node will elongate to the new head node.

*Intersection algorithm:* (i) retrieve x,y coordinates of head node, (ii) search through Tracker for x,y coordinates near head node, (iii) check if head node intersects with coordinates near head node, (iv) if head node does intersect, return True. Otherwise, return False.

When the bifurcation event is triggered (Fig 3D), two new head nodes will be created from the current head node (Fig 3E). Two new angles are randomized, centered about the angle of the current head node. Then the elongation process is performed, starting from the two new angles. The elongation process is described in more detail later.

*Bifurcation algorithm:* (i) use biased Random Value Function to select a value *p*, (ii) retrieve current angle position of head node θ, (iii) create two new head nodes with angle position p+θ and p−θ, (iii) run elongation algorithm with new head nodes.

The elongation algorithm is how the random walk function is performed. From the current head node, a step of size 1 is taken. The x and y coordinates are determined by cos(θ) and sin(θ), respectively.

*Elongation algorithm:* (i) retrieve x,y coordinate $\left(x_{0},y_{0}\right)$ and current angle $\alpha_{0}$ of the current head node, (ii) use biased random value function with input $\alpha_{0}$ to determine new angle θ, (iii) new x,y coordinates are defined by $\left(x_{0}+cos(\theta ),y_{0}+sin(\theta )\right)$, (iv) check if new x,y coordinates intersect another branch the Intersection algorithms, (v) if its true the new x,y coordinates intersect, terminate the branch. Otherwise, elongate from the current head node to new x,y coordinates. The current head node will become a body node, and the new x,y coordinates will become a new head node. The new head node is added to the Tracker.

Prior to using the biased random walk model, a preformed random walk network with no bias was used. This is to simulate prepubertal growth, similar to the glands we are analyzing. Bias was introduced after the preformed network was formed by performing the biased random walk model on the head nodes of the preformed network. All simulations used the same preformed network. Analysis was conducted on the biased growth, not including the preformed network.

## 3 Results

### 3.1 Application of uniaxial force did not affect the area of the ductal network when uniaxial force was applied from 5-7 weeks

**Fig 4:**
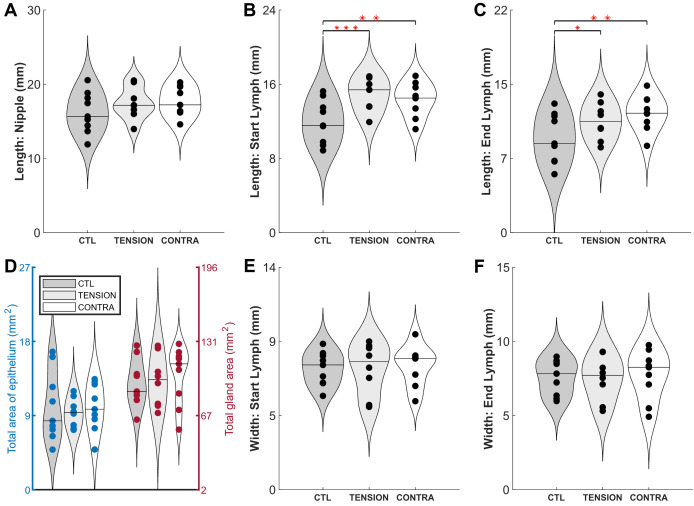
Primary experiment morphometrics. Shows morphometric significance with outliers removed. A-C) compares the length from nipple to distal tip, start of lymph node to distal tip, and end of lymph node to distal tip. D) compares the total epithelial area and the total gland area spanned by the ductal network. E-F) compares the width from the start of the lymph node to the distal tip and the end of the lymph node to the distal tip. Horizontal lines through the violin plot represent the median of the data. *p < 0.1, **p < 0.05, ***p < 0.01.

The cross-sectional area of the ductal network and the whole mammary gland was used to estimate their size. The total cross-sectional area of the epithelial ductal network for TENSION and CONTRA glands was compared to CTL to determine if the uniaxial force altered the area of the epithelial ductal network. Similarly, the total area of the mammary gland was also compared to determine if uniaxial and contralateral forces altered the overall size of the mammary gland. Analysis of mammary glands from the first experiment, when the uniaxial force was applied from 5-7 weeks of age, found no statistically significant difference in the cross-sectional area for the mammary glands in TENSION vs. CTL (*p* = 0.52) and in CONTRA vs. CTL (*p* = 0.27). And no significant difference in the area of the epithelial ductal network ($p_{TENSION}=0.52,\;p_{CONTRA}=0.33$; Fig 4D). Additional morphometrics such as curvature and number of perimetral endbuds were not significant (S6 Fig and S1 Text).

### 3.2 Uniaxial force significantly increased the overall length of the epithelial ductal network for the TENSION glands when uniaxial force was applied from 5-7 weeks

The length of the epithelial network from the beginning of the lymph node (the side closer to the nipple) to the distal tip was significantly longer in TENSION (*p* = 0.006) and CONTRA (*p* = 0.03) glands compared to CTL, with or without outliers removed (Fig 4B). At 5 weeks of age, the ductal network in the abdominal four mouse mammary gland has barely reached the start of the lymph node; therefore, the length of the epithelial network from the beginning of the lymph node to the distal tip gives us a good estimate of how the uniaxial force applied from 5-7 weeks of age affected the epithelial network. The length from the end of the lymph node (the side further from the nipple) to the distal tip was also significantly longer in CONTRA glands than CTL (*p* = 0.02) and longer in TENSION glands compared to CTL with 90% confidence (*p* = 0.08), with or without outliers removed. Therefore, the increased length for the ductal network in the TENSION and CONTRA glands does not correspond to an increase in the cross-sectional area of the epithelium tissue and the total mammary gland. The uniaxial and contralateral forces did not alter the width of the epithelial ductal network; therefore, the increased length of the ductal network observed in the TENSION and CONTRA glands did not correspond to an increase in the aspect ratio (i.e., the ratio of the length to the width) of the epithelial ductal network. We attribute this to the uniaxial and contralateral forces changing the shape of the fat pad, but not the total area. The change in shape may allow for branches to grow longer without sacrificing surface area.

### 3.3 Uniaxial force altered the orientation of the ducts in the TENSION and CONTRA glands when uniaxial force is applied from 5-7 weeks

**Fig 5:**
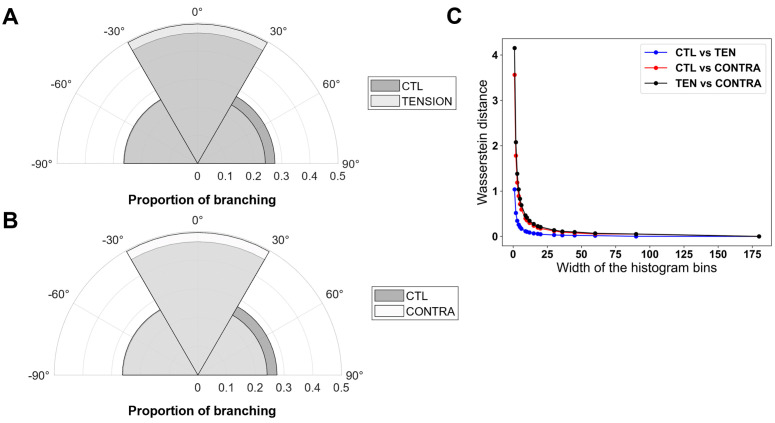
Primary experiment: Comparison of the angular distribution of the bulk of the ductal network for TENSION, CONTRA, and CTL. Visual differences in angle distribution for TENSION versus CTL and CONTRA versus CTL are shown in (A) and (B), respectively. Here, the bin size is set at $60^{\circ }$ angle. (C) The graph of Wasserstein distance against bin size shows how the degree of similarity in angle distribution between the different groups is dependent on the bin size.

The orientation of the individual ducts in the ductal network determines the spatial connectivity and overall shape of the ductal network, and may potentially affect the development of subsequent milk-producing glandular tissue. Given the importance of ductal orientation, we sought to determine if the angular positions of the ducts were affected by the uniaxial force. Fig 5A and 5B shows the distribution of the ducts using histograms with bins of $60^{\circ }$ width. The TENSION and CONTRA glands had a higher proportion of branching within $\left[-30^{\circ },30^{\circ }\right]$ and a lower proportion of branching within $\left[30^{\circ },90^{\circ }\right]$. Reducing the bin size of the histogram allowed us to determine smaller range of angles that contribute more to the observed changes in ductal network orientation between TENSION versus CTL and CONTRA versus CTL (see Table B in S1 Text). Using a $20^{\circ }$ bin size, we found that CTL had a significantly larger proportion of branching in the $\left[50^{\circ },69^{\circ }\right]$ range than TENSION (Table B in S1 Text). By reducing the bin size to $5^{\circ },$ we obtained granular information of angle ranges where CTL had a significantly larger proportion of branching angles than TENSION. When we used the $5^{\circ }$ bin size, we found that CTL had a slightly larger proportion of branching than TENSION in three of the four bins between $\left[50^{\circ },69^{\circ }\right]$ (i.e., $[50^{\circ },54^{\circ }],$ $\left[60^{\circ },64^{\circ }\right]$ and $\left[65^{\circ },69^{\circ }\right]$; Table B in S1 Text). By combining the analysis from the $5^{\circ }$ bin size to the $20^{\circ }$ bin size, we inferred that the contributions from $[50^{\circ },54^{\circ }],$ $\left[60^{\circ },64^{\circ }\right]$ and $\left[65^{\circ },69^{\circ }\right]$ angle ranges together made CTL have significantly more branching than TENSION in the $\left[50^{\circ },69^{\circ }\right]$ angle range. Similar observations were observed with or without outliers removed (Table B and Table E in S1 Text).

Note that if the bin size is very large (say $180^{\circ }$), then one will not be able to capture the differences between TENSION, CONTRA, and CTL ductal networks. On the other hand, if the bin size is too small (say $1^{\circ }$), one will only learn that each ductal network is different. In order to select a good bin size for comparing the histograms, the similarity in the distribution of the angular positions for TENSION vs. CTL and CONTRA vs. CTL for different bin sizes was computed. The similarity in the distribution of the angular positions for TENSION vs. CTL and CONTRA vs. CTL was measured using a novel approach that utilizes the Wasserstein distance. Wasserstein distance clearly captures the expected dynamics for how the similarity between the angle distributions would change with the histogram’s bin size (Fig 5C). A low value of Wasserstein distance indicates a high similarity between the distributions, and a high value of Wasserstein distance indicates a high degree of dissimilarity between the distributions (Fig 5C). The graph of Wasserstein distance against bin size resembles a visual tool called an elbow plot that is often used in cluster analysis and other machine learning algorithms to determine the optimal number of clusters [52]. In particular, the elbow plot identifies the point at which adding more clusters does not significantly improve the model performance, indicating a diminishing return. Using a similar idea as often used for elbow plots, a bin size of $5^{\circ }$ was selected because it gives a value for Wasserstein distance close to the elbow of the graph in Fig 5C.

**Fig 6:**
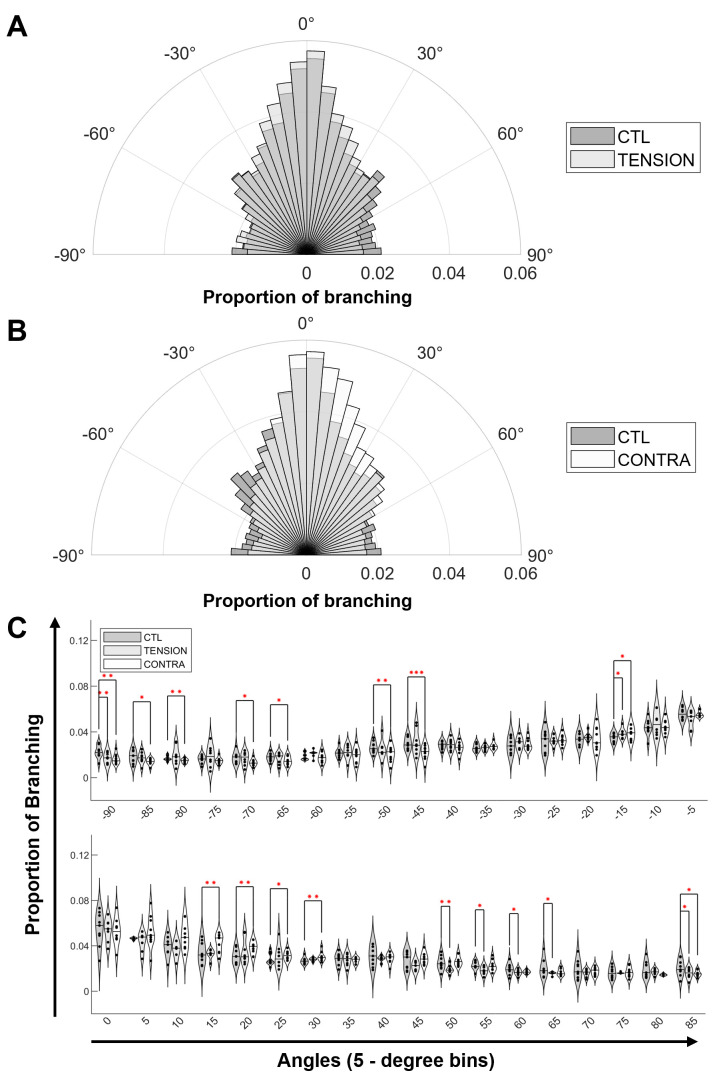
Angle distributions for primary experiments. (A)-(B) Angle distribution for the bulk of the ductal network from the start of the lymph node to the distal tip. In (A)-(C), the bin size is set at $5^{\circ }$ angle. (C) Angle distributions from the start of the lymph node to the distal tip with outliers removed. Angle value on the x-axis represents the lower bound of the bin.

Fig 6 shows a visual comparison of the angle distribution for the ductal network for TENSION versus CTL and CONTRA versus CTL using $5^{\circ }$ angles for the bin size, after outliers were removed. Consistent with the angle distribution with $60^{\circ }$ bin size, we see here that TENSION glands consistently had a higher proportion of ductal regions with smaller angular position (i.e., between [$-35^{\circ },35^{\circ }$], Fig 6A). A different visual pattern evolved for the angle distribution for CONTRA versus CTL (Fig 6B). Fig 6B indicates that the higher proportion of angular regions in $\left[-30^{\circ },90^{\circ }\right]$ angles was due to a high proportion of ductal regions existing in the $\left[-5^{\circ },40^{\circ }\right]$ degree range. The CONTRA glands experienced the uniaxial force on one side of its gland, that is, on the side adjacent to the TENSION gland. The higher proportion of smaller angles was only evident in one direction of the ductal network of CONTRA vs. CTL, towards the positive side of the gland (Fig 6B). Therefore, Fig 6A and 6B suggests that the uniaxial force altered the orientation of the ducts in the TENSION and CONTRA glands.

Analysis of the distribution of angles for each individual ductal network showed that the angle distributions were altered by differing application of forces, TENSION and CONTRA, relative to CTL (Fig 6C). The TENSION glands tended towards a higher proportion of branching compared to CTL at $-15^{\circ }$ to $-11^{\circ }$ (*p* = 0.09). The CTL glands had a significantly higher proportion of branching compared to TENSION glands within -90° to -86° and 50° to 54°, and tended towards significance between $55^{\circ }$ to $69^{\circ }$ and $85^{\circ }$ to $89^{\circ }$ range of angles. In contrast, CONTRA had a significantly higher proportion of ductal branching compared to CTL within $15^{\circ }$ to $24^{\circ }$ and $30^{\circ }$ to $34^{\circ }$ and tended towards significance between $-15^{\circ }$ to $-11^{\circ }$ and $25^{\circ }$ to $29^{\circ }$ CTL had a significantly higher proportion of ductal branching within $-90^{\circ }$ to $-86^{\circ }$, $-80^{\circ }$ to $-76^{\circ }$, and $-50^{\circ }$ to $-41^{\circ }$ compared to CONTRA. CTL glands also tended to have a higher proportion of branching within $-85^{\circ }$ to $-81^{\circ }$, $-70^{\circ }$ to $-61^{\circ }$, and $85^{\circ }$ to $89^{\circ }$. Thus, the uniaxial force biased ductal orientation differently in CONTRA compared to the TENSION glands, indicating that the direction of the applied force is important for controlling the orientation of the ducts.

Considering that the orientation of daughter ducts is in general, determined by the branching angles of daughter ducts from their parent duct, it was hypothesized that the increased length of the ductal network for the TENSION and CONTRA glands may have resulted from changes in the branching angle of the ducts. To test this hypothesis, ductal network formation was simulated using a biased branching and annihilating random walk model, and the lengths of the ductal networks were analyzed to determine if varying the angle of branching can alter the length of a ductal network.

### 3.4 Biased and annihilating random walk model predicts that the increased length of the ductal network in the TENSION glands was due to the existence of a smaller branch angle for ductal bifurcation in TENSION compared to CTL gland

**Fig 7:**
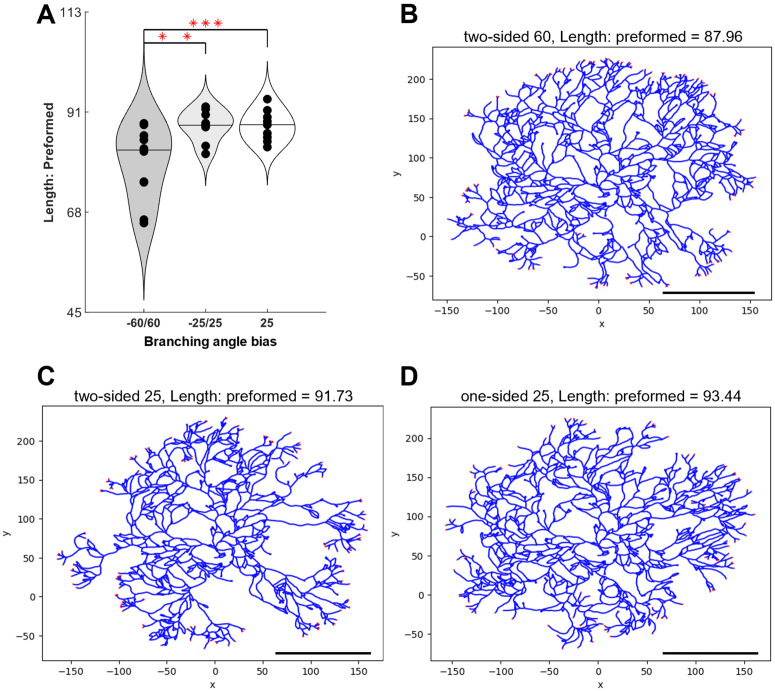
Biased and annihilating random walk simulations mimicking primary experiment. (A) shows the significant difference between the length of the simulated CTL (B), TENSION (C), and CONTRA (D) glands; (B, C, & D) shows a sample simulated CTL, TENSION, and CONTRA glands respectively. The glands were formed with a preformed network of 100 generations with no bias. Following the preformed network, bias was introduced for 100 additional generations. The length (black bar) is taken from the end of the preformed network to the end of the simulation. In (A), the y-axis is truncated to emphasize differences within the observed range.

The biased and annihilating random walk model was simulated for at least 8 instances per bias value. To simulate TENSION values from the bulk data in Fig 6A were used. For CTL, a combination of the bulk and individual values was used (Fig 6A and 6C). These values include $-60/60^{\circ },-75/75^{\circ },-80/80^{\circ },$ and $-90/90^{\circ }$ for CTL and $-15/15^{\circ },-20/20^{\circ },-25/25^{\circ },$ and $-30/30^{\circ }$ for TENSION. The random walk model was run for 100 generations per bias value. The length of the biased ductal networks was computed and compared to those obtained from the laboratory experiments. The results from the simulations predict that smaller angles of ductal bifurcation can significantly increase the length of the ductal network (Fig 7).

Further analysis for one-sided bias was conducted, where the simulation was given only one angle, rather than a positive and negative angle of the same distance from $0^{\circ }$. For CONTRA glands, $15^{\circ },20^{\circ },25^{\circ },$ and $30^{\circ }$ angles were used. Simulations from the one-sided bias were also compared to the two-sided bias, where a positive and negative angle of the same distance from 0° was used as bias. Comparison of the simulations from the one-sided bias with the two-sided biases indicates that small branching angles, one- or two-sided, result in significantly longer ductal networks than ductal networks that prefer to branch at larger branching angles (Table 1). The line between small and large branching angles appears to be at or above $35^{\circ }$. The results also show no difference in one- and two-sided bias lengths of the same positive angle value.

**Table 1:** Comparison of the one-sided and two-sided bias simulations of the biased and annihilating random walk model. The values in the Table are p-values from the one-sided Mann-Whitney U test with outliers removed. The p-values indicate the statistical differences in the ductal network when testing if the bias angle in row is significantly greater than that in the corresponding column.

		One-sided bias			Two-sided bias							
		20	25	30	60	75	80	90	15	20	25	30
**One-sided bias**	**15**	0.07	0.10	0.04	0.0008	0.0001	0.0008	0.0001	0.19	0.03	0.14	0.01
	**20**		0.66	0.46	0.04	0.01	0.02	0.02	0.85	0.44	0.66	0.27
	**25**			0.35	0.01	0.0002	0.004	0.0005	0.55	0.19	0.56	0.12
	**30**				0.02	0.0003	0.006	0.002	0.70	0.27	0.77	0.20
**Two-sided bias**	**60**					0.08	0.27	0.14	0.99	0.84	0.99	0.93
	**75**						0.64	0.70	1.0	0.99	1.0	1.0
	**80**							0.60	1.0	0.9	1.0	0.99
	**90**								1.0	0.99	1.0	1.0
	**15**									0.14	0.45	0.10
	**20**										0.81	0.48
	**25**											0.10

### 3.5 Secondary experiments reveal patterns consistent with those for the primary experiment

In order to better understand the impacts of application of uniaxial force in relation to the developmental stage of the mammary gland, a second experiment was conducted. For this experiment, a uniaxial force was applied from 5-6 weeks of age. Half the mice in the CTL and experimental groups were euthanized for mammary morphological analysis. In the remaining mice, the uniaxial force was removed from the experimental group at 6 weeks, and then 7 days later, mammary glands were collected from CTL and TENSION/CONTRA mice at 7 weeks of age.

**Fig 8:**
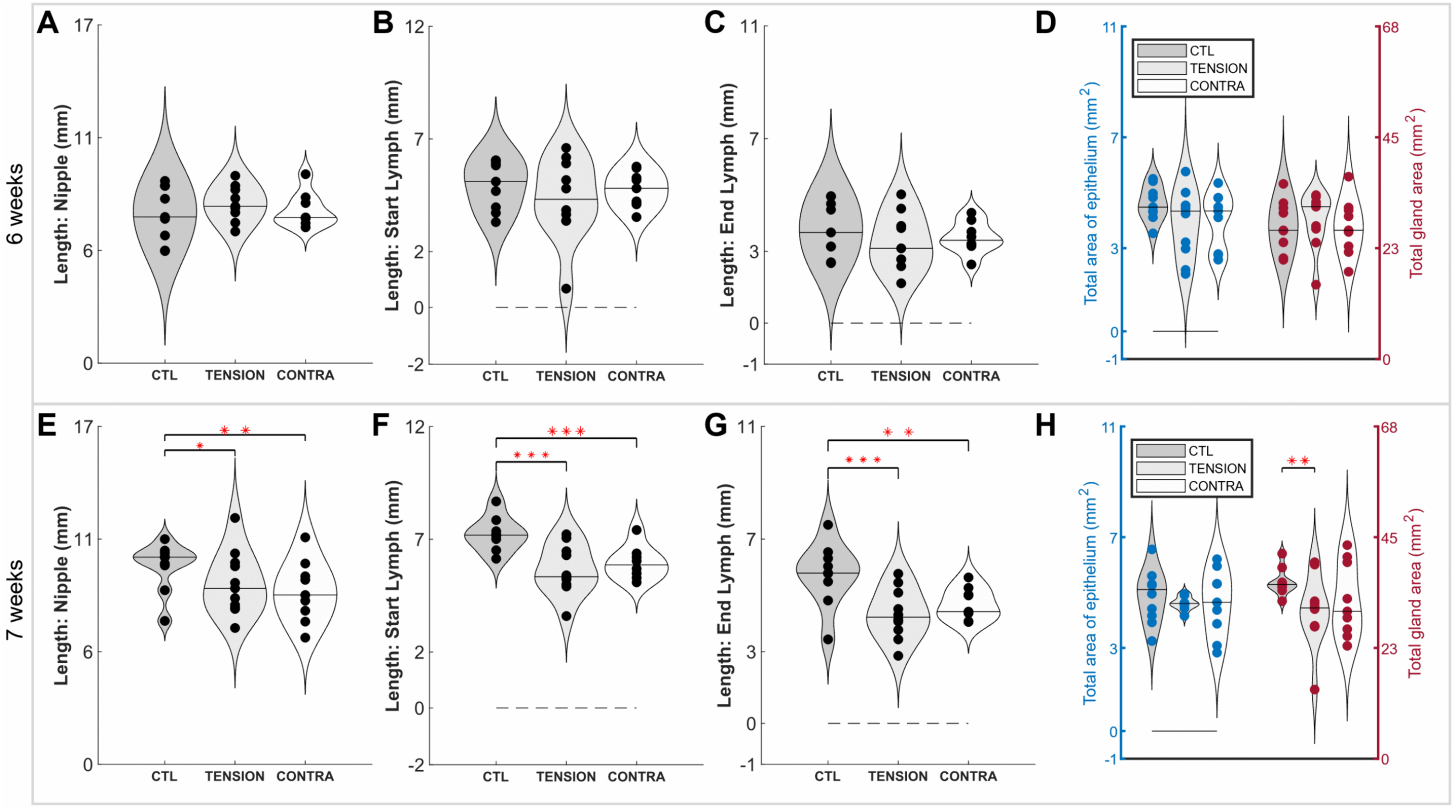
Secondary experiment morphometrics. Shows morphometric significance with outliers removed. A-C) compares the length of the secondary 6 week data from nipple to distal tip, from start of lymph node to distal tip, and from end of lymph node to distal tip. D) compares the epithelial area and total gland area spanned by the ductal network of the secondary experiment for mice at age 6 weeks, E-G) compares the length from nipple to distal tip, start of lymph node to distal tip, and end of lymph node to distal tip for the secondary experiment for mice at age 7 weeks. H) compares the epithelial area and total gland area spanned by the ductal network of the secondary experiment for mice at age 7 weeks.

Analysis of ductal network length, total epithelial area, and total gland area spanned by the ductal network at 6 weeks revealed no differences among the CONTRA, TENSION, and CTL (Fig 8A-8D). Analysis of the glands at 7 weeks, which was 1 week after removal of uniaxial force, found that the ductal networks of CONTRA and TENSION glands, measured from the beginning of the lymph node (the side closer to the nipple) to the distal tip, were shorter than CTL glands (Table 2, p < 0.05). At age 6 weeks, the majority of the ductal network has barely passed the lymph node, so comparing the difference in length from the beginning of the lymph node to the distal tip at week 7 is important.

**Table 2:** Shows all p-values that adjusted in significance level when outliers were removed for key morphometrics.

Experiment	Morphometric	Significance direction	p-value with outliers	p-value after removal of outliers
Primary	Length: Start Lymph	TENSION > CTL	0.037	0.006
Secondary (mice aged 6 weeks)	Width: End Lymph	TENSION > CTL	0.33	0.098
Secondary (mice aged 7 weeks)	Length: Nipple	TENSION < CTL	0.15	0.06
	Length: Nipple	CONTRA < CTL	0.096	0.035
	Length: Start Lymph	TENSION < CTL	0.14	0.003
	Length: Start Lymph	CONTRA < CTL	0.047	0.005
	Length: End Lymph	TENSION < CTL	0.057	0.004
	Total Gland Area	TENSION < CTL	0.065	0.01

We also compared the ductal network length from the end of the lymph node (on the side further from the nipple) to the distal tip and from the nipple to the distal tip at 7 weeks. Measurements of ductal network length taken from the end of the lymph node to the distal tip found that TENSION glands were significantly shorter than CTL (Table 2, p = 0.004), indicating that the portion of the ductal network that was past the lymph node was on average shorter than those in CTL. Similarly, in the CONTRA glands, the ductal network length from the end of the lymph node to the distal tip was also found to be significantly shorter than CTL (p = 0.01). The median length for CTL when measured from the end of the lymph node to the distal tip for glands at week 6 was 3.28 mm compared to week 7 at 5.43 mm. For TENSION, the median length increased from 2.7 to 3.93 mm from week 6–7, whereas the median length for CONTRA grew from 2.87 to 4.04 mm. This means CTL glands grew approximately 2.15 mm between weeks 6 and 7, whereas TENSION and CONTRA only grew 1.23 and 1.17 mm, respectively. To see a millimeter scale bar, see Fig 2.

**Fig 9:**
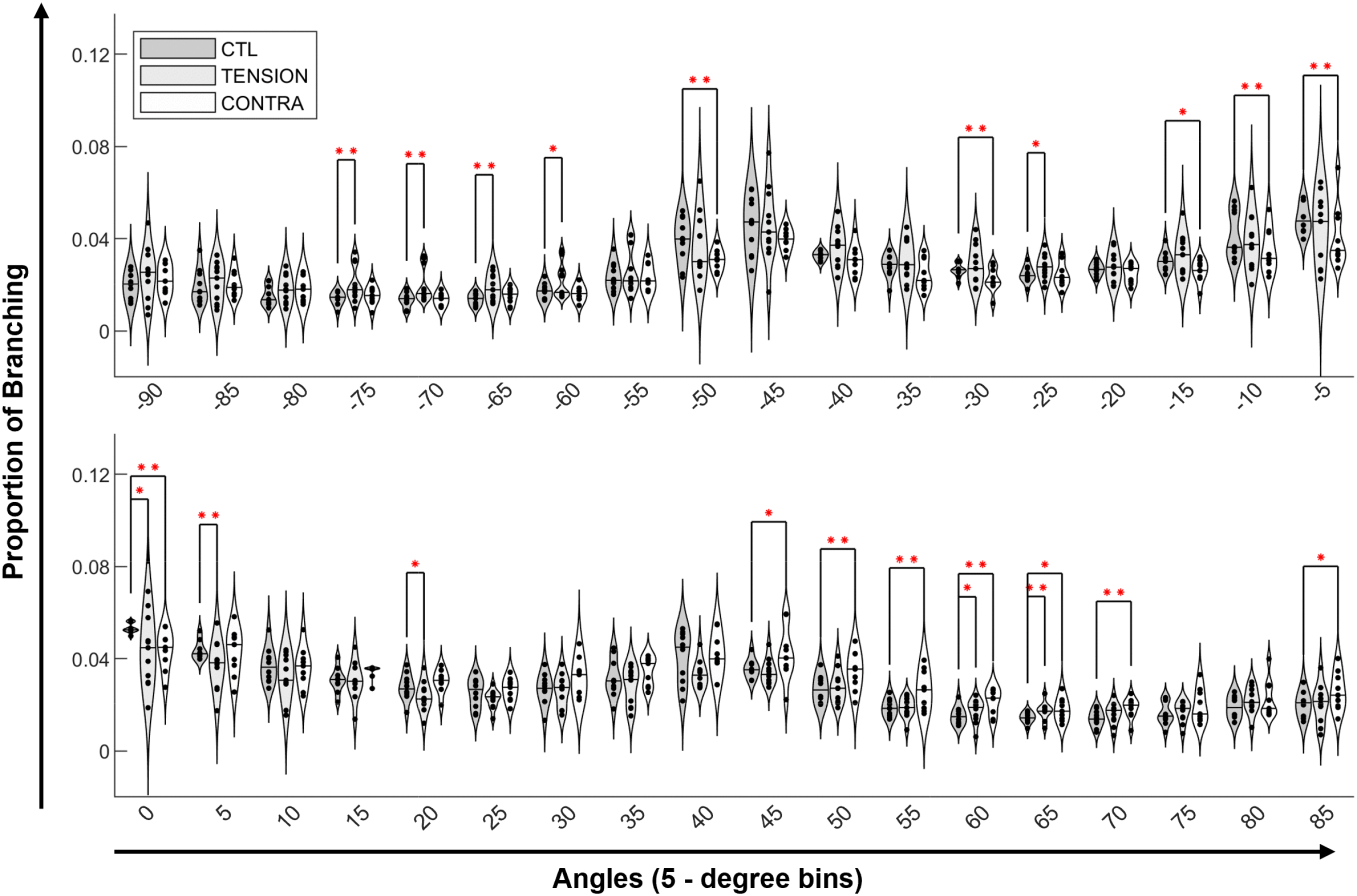
Angle distributions for secondary experiment for mice aged 7 weeks. Angle distributions from the end of the lymph node to the distal tip with outliers removed. Angle values on the x-axis represents the lower bound of the bin.

When the ductal network length was measured from the nipple to the distal tip, only CONTRA was significantly shorter than CTL at 95% confident interval (Table 2, p = 0.035). Though not statistically significant, the ductal network length measured in the TENSION glands from the nipple to the distal tip was, on average, slightly shorter than CTL. We also found a greater proportion of larger branching angles in the TENSION and CONTRA glands compared to CTL from the end of the lymph node to the distal tip in the 7 week experiments (Fig 9). The larger branching angles are present on the positive side of CONTRA glands, consistent with Fig 6 for the primary experiment. This suggest that shorter ductal network lengths are consistent with greater proportion of larger branching angles. Additional morphometric measures, such as width, curvature, and number of perimetral endbuds can be found in S7 Fig and S1 Text.

The secondary experiments for the mice 7 weeks of age suggest that after constant uniaxial and contralateral forces are removed, there may be a refractory response, leading to shorter length development and larger branching angles.

**Fig 10:**
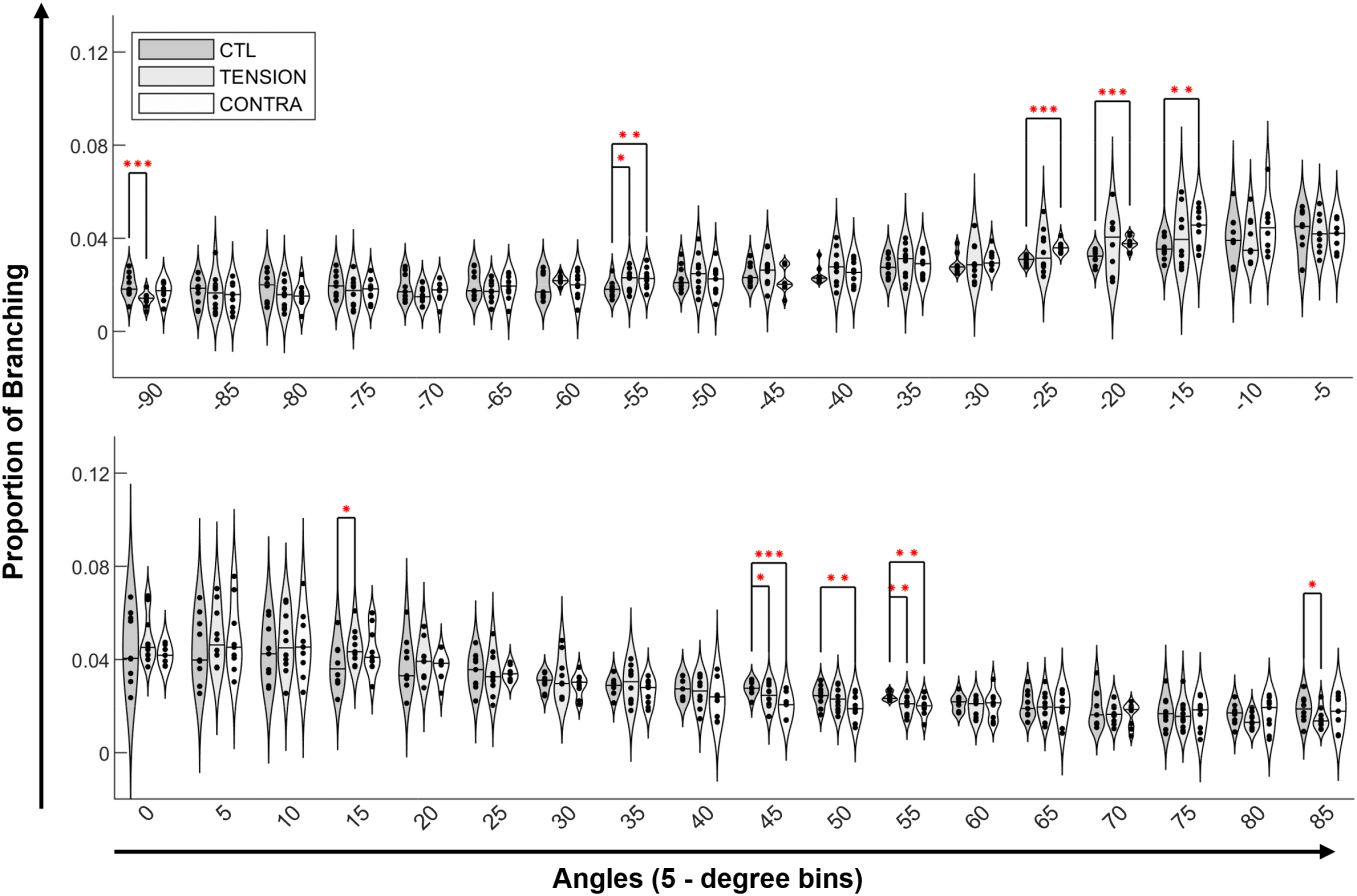
Angle distributions for secondary experiment for mice at age 6 weeks. Angle distributions from the nipple to the distal tip with outliers removed. Measurements for the ductal network length were taken from the nipple to the distal tip since the ductal network had barely passed the lymph node at week 6. Angle value on the x-axis represents the lower bound of the bin.

**Fig 11:**
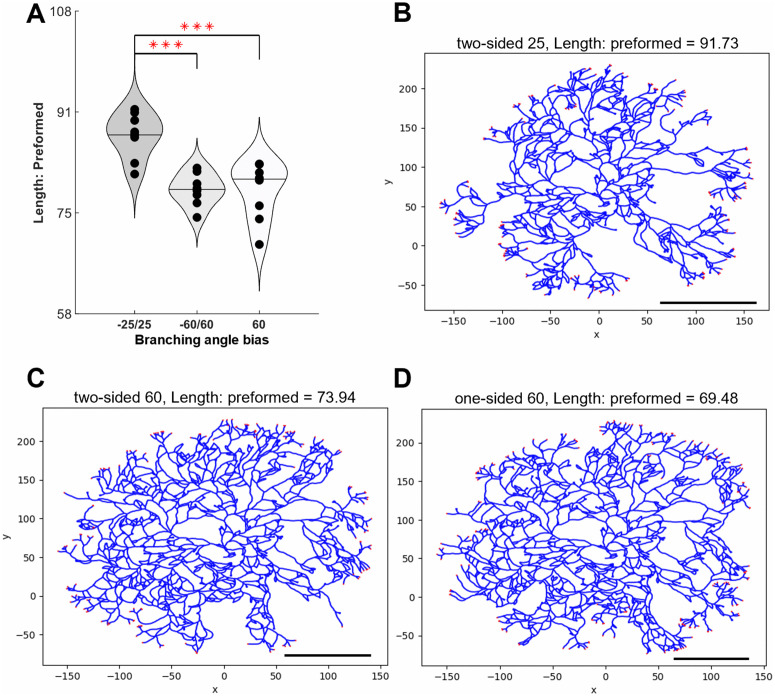
Biased and annihilating random walk simulations mimicking the 7-week-old mice from the secondary experiment. (A) shows the significant difference between the length of the simulated CTL (B) compared to simulated TENSION (C) and CONTRA (D) gland from 7 weeks old mice from the secondary experiment (B) and CTL (C-D) glands; (B, C, & D) shows a sample simulated 7-week-old mice from the secondary experiment CTL, TENSION, and CONTRA glands respectively. The length (black bar) is taken from the end of the preformed network to the end of the simulation. In (A), the y-axis is truncated to emphasize differences within the observed range.

Random walk simulations were performed to recreate the smaller branching angles observed in the secondary experiments for the mice aged 6 weeks (Fig 10), followed by larger branching angles observed in the mice aged 7 weeks (Fig 9). To recreate this, a biased random walk model at $-30/30^{\circ }$ was run for 50 generations on top of the preformed network. This value was used as $-30/30^{\circ }$ is around the boundary of what defines a small branching angle. The one-sided $30^{\circ }$ was not used as Table 1 reveals there is no difference between one-sided and two-sided angle differences for this angle. After, additional one- and two-sided bias was introduced using angles observed in Fig 9 and Table D in S1 Text to mimic CONTRA and TENSION branching angles, respectively. These results were compared to the random walk experiments from the primary experimental data. Results show that when you apply a small branching angle, followed by a large branching angle, the length will be smaller than simply using a small branching angle (Fig 11 and Table 3). This is consistent with the secondary experiments, where TENSION and CONTRA were characterized by larger branching angles, and CTL were characterized by smaller branching angles for mice aged 7 weeks.

**Table 3:** Comparison of simulations of biased and annihilating random walk models using information on angle measurements for the 7-week mice from the secondary experiment. The p-values in the Table indicate the statistical differences in the ductal network when testing if the bias angle in each row is significantly greater than those in each column, after outliers are removed. The columns denoted *Bias for secondary experiment for mice aged 7 weeks* show the simulation results for two-sided bias for 50 generations, on top of a biased preformed network at -30/30 for 50 generations and the initial unbiased preformed network.

		Bias for secondary experiment for mice aged 7 weeks							
		-60/60	-70/70	-80/80	-90/90	60	70	80	90
**One-sided bias**	**15**	0.0001	0.0001	0.0001	0.0001	0.0001	0.0001	0.0001	0.0001
	**20**	0.016	0.006	0.021	0.021	0.008	0.021	0.012	0.008
	**25**	0.0002	0.0002	0.0002	0.0002	0.0002	0.0003	0.0002	0.0002
	**30**	0.0006	0.0006	0.0003	0.0006	0.001	0.0006	0.0006	0.0003
**Two-sided bias**	**60**	0.13	0.07	0.13	0.15	0.15	0.15	0.11	0.071
	**75**	0.73	0.42	0.66	0.70	0.73	0.73	0.59	0.46
	**80**	0.73	0.23	0.70	0.77	0.54	0.66	0.58	0.34
	**90**	0.73	0.14	0.73	0.73	0.57	0.65	0.48	0.12
	**15**	0.0004	0.0002	0.0001	0.0004	0.0006	0.0.0002	0.0002	0.0001
	**20**	0.04	0.01	0.03	0.02	0.04	0.04	0.01	0.007
	**25**	0.0006	0.0006	0.0003	0.0006	0.0006	0.0003	0.0003	0.0003
	**30**	0.003	0.003	0.0019	0.003	0.005	0.005	0.002	0.0006

## 4 Discussion

Identifying the mechanisms that regulate the orientation of the ductal branches is an important step towards unraveling the complex interactions that coordinate the formation of the mammary ductal network during puberty. A crucial step for elucidating how the orientation of the epithelial ductal branches is specified is to identify the mechanisms that modulate or regulate the branch orientation *in vivo*. Biomechanical forces originating from cell-cell and cell-ECM interactions actively remodel the tissue mechanical environment, modulating mechanotransduction pathways that regulate cell migration, tissue organization, and morphogenesis. In this study, we investigated if an external uniaxial force applied during puberty can regulate the orientation of epithelial ducts in the ductal network. In the first experiment, uniaxial forces were applied continuously to the right abdominal number four mouse mammary glands (n = 10; TENSION) from 5-7 weeks of age, during pubertal formation of the epithelial ductal network. The uniaxial force was applied by pulling and adhering the skin around the nipple of the right abdominal number four mammary gland. Using a combination of in vivo experiments and computational modeling, we demonstrated that a uniaxial force applied to mice mammary glands for two weeks during pubertal development increases the length of the ductal network and modifies the orientation of ducts in the ductal network. The cross-sectional area of the ductal networks that were exposed to the uniaxial and contralateral force (i.e., the TENSION and CONTRA glands) was similar to CTL, which indicates that increased length of the ductal network does not correspond to an increase in the cross-sectional area of the ductal network. Therefore, the applied forces changed the ductal network in order for TENSION and CONTRA to have a longer ductal network while having a similar cross-sectional area as CTL.

Given the importance of ductal orientation, we sought to determine if the angular positions of the ducts were affected by the uniaxial force. The proportion of ducts in each degree angle was used as a measure for the orientation of the ducts in the TENSION, CONTRA, and CTL glands. We introduced a novel approach that utilizes Wasserstein distance metrics to identify an optimal bin size for comparing the distributions of ductal angles in TENSION and CONTRA glands to CTL. The proportion of ducts at different angular regions in the ductal network for TENSION and CONTRA was compared to CTL, and we were able to determine how the orientation of ducts differs in TENSION and CONTRA versus CTL. CTL glands had a greater proportion of branching within the larger angles ranging from $\pm 90^{\circ }$to $\pm 50^{\circ }$. While we do not see much significance in smaller branching angles for TENSION compared to CTL at an individual level, we do see that TENSION has a larger proportion of branching at smaller angles compared to CTL at a bulk level (Fig 6A). We also see that TENSION has a smaller proportion of branching at larger angles compared to CTL at both an individual and a bulk level. We can hypothesize that the uniaxial force causes the change in branching angle to be spread over a range of small branching angles, around -35° to 35°. This can also be seen in the 60-degree bin bulk data in Fig 5. As such, smaller branching angles will alter the direction of growth more towards the distal tip, rather than the positive and negative sides of the gland.

CTL had a higher proportion of ductal branching within -90° to -86°, -80° to -76°, and -50° to -41° compared to CONTRA. In contrast, CONTRA had a higher proportion of ductal branching compared to CTL from 15° to 24° and 30° to 34°. Therefore, the contralateral force in the CONTRA glands also biases the orientation of ducts towards the smaller angle ranges, but only in the positive range. Thus, the uniaxial force biased ductal orientation differently in CONTRA compared to the TENSION glands. This implies that the direction of the applied force influences the orientation of the ducts.

Furthermore, we hypothesized that the increased length in the ductal network for the TENSION and CONTRA glands may have been due to changes in the orientation of the ducts compared to CTL. Since the orientation of daughter ducts is, in general, associated with the angle of branching of daughter ducts from their parent duct, we tested this hypothesis by examining how variation in the ductal branch angles affects the length of the ductal network, using a biased branching and annihilating random walk model. Results from the biased branching and annihilating random walk simulations predict that the increased length of the ductal network in the TENSION glands may have been caused by alterations in branching angles. In particular, narrow ductal branch angles led to an increase in the length of the ductal network. Based on our findings, we concluded that the uniaxial force potentially caused an increase in the length of the ductal network of the TENSION glands by modifying the orientation of the ductal branches.

For experiment two, the uniaxial force was applied from 5-6 weeks, and glands from half the mice (n = 10/group TENSION and control, CTL) were collected. In the remaining mice, the application of force was removed at 6 weeks from the TENSION group, and then glands were collected at 7 weeks of age n = 10/group TENSION and CTL). This secondary experiment reveals patterns consistent with those of the primary experiment. The branching angles of the entire glands were significantly impacted by application of uniaxial force from 5-6 weeks. At 6 weeks, TENSION and CONTRA glands had a larger proportion of smaller branching angles than CTL and CTL had a greater proportion of larger branching angles compared to TENSION and CONTRA (Fig 10). Analysis of ductal network length at 6 weeks found no difference between CONTRA, TENSION, and CTL (Fig 8A-8C). Analysis of the glands at 7 weeks, which was 1 week after removal of uniaxial force, found the ductal networks of CONTRA and TENSION glands, measured from the beginning of the lymph node to the distal tip, were shorter than CTL glands (Table 2, p < 0.05). At age 6 weeks, the majority of the ductal network has barely reached the lymph node, so comparing the difference in length from the start of the lymph node to the distal tip at week 7 is important.

We also found a greater proportion of larger branching angles in the TENSION and CONTRA glands compared to CTL from the end of the lymph node to the distal tip (Fig 9) for the mice aged 7 weeks in the secondary experiments. The larger branching angles are present on the positive side of CONTRA glands, consistent with Fig 6 for the primary experiment. This suggests that shorter lengths are consistent with large branching angles. The secondary experiment for the mice at 6 weeks of age suggests that constant uniaxial and contralateral forces are consistent with smaller branching angles. However, after these forces are removed, there may be a refractory response leading to shorter length development and larger branching angles.

Random walk simulations were performed to recreate the smaller branching angles observed in the secondary experiments for the mice aged 6 weeks, followed by larger branching angles observed in the mice aged 7 weeks. Results show that when you apply a small branching angle, followed by a large branching angle, the length of the ductal network will be smaller than simply using a small branching angle (Fig 11 and Table 3). This follows from the secondary experiments, where TENSION and CONTRA were characterized by larger branching angles, and CTL were characterized by smaller branching angles for mice aged 7 weeks.

Studies on mouse embryonic lung morphogenesis, in which mechanical forces were applied by increasing intraluminal pressure, have shown that mechanical forces can accelerate lung growth and increase by 2-fold the average number of new epithelial branches between 24 and 48 h of culture [53]. Though the total number of epithelium branches in the embryonic lung was doubled, the diameter of the branches was reduced by 30%, and the global size of the lungs remained similar to the control ones [53]. This shows that mechanical forces can change the organization and dimensions of the epithelial branches in the mammary glands and lungs, while the overall size of the epithelial branched network stays unchanged.

This study raises new questions concerning the mechanism by which the uniaxial force regulates the orientation of the ducts. That is, whether the changes in ductal orientation were orchestrated by mechanisms such as pure physical interactions between the ECM and ductal cells, molecular factors, a combination of both physical and molecular factors, or perhaps through entirely different mechanisms. Given that a uniaxial force applied to a tissue can cause a uniaxial strain, resulting in collagen reorganization in the direction of the uniaxial force, and changes in collagen organization can alter mechanical signaling, influence cell adhesion, migration, morphology, and differentiation [30,31], which are key processes involved in branching morphogenesis [32]. Future work may focus on understanding how uniaxial force impacts cell behavior and collagen fiber orientation during branching morphogenesis. This may yield new insights into how the tension force generated by interactions between the ductal cells and its surrounding ECM affects cell behavior and the process of mammary branching morphogenesis. Although we applied a force (i.e., a pull that caused the tissue to deform), we had no way to measure its magnitude. In addition, the mammary gland is embedded within a heterogeneous, multilayered environment, and thus the transmission of force within the tissue is unlikely to be uniform or strictly uniaxial. Therefore, the applied force should be interpreted as an approximation describing the directionality of the perturbation rather than a fully characterized mechanical state. Future work may aim to develop approaches to quantify the applied force and to investigate how its magnitude and distribution vary with changes in ductal morphology.

There are several limitations of this study. An important one is that the nipple region was not consistently captured across all carmine-stained images. In cases where the nipple was not clearly visible, the approximate outflow point was inferred by following the direction of the ducts. While this approach provides a reasonable estimate, it introduces some uncertainty in measurements defined relative to the nipple, particularly given that the observed differences are on the millimeter scale. To mitigate this, measurements were performed independently by two individuals and assessed for consistency, and we did not rely solely on nipple-based measurements. Instead, we also quantified ductal lengths using alternative anatomical reference points, such as the start and end of the lymph node. Nevertheless, the absence of a clearly identifiable nipple in some images should be considered when interpreting small differences in ductal network length.

Another limitation is that CONTRA glands were not analyzed with a left control group, which precludes a direct assessment of baseline left-right variability of mammary gland response. Therefore, we cannot formally exclude a contribution of intrinsic laterality to the observed differences, although the consistency of the effects across experimental conditions supports an interpretation driven primarily by the applied uniaxial force. We note that the use of a common reference (the right CTL mammary glands) across all experimental conditions provides an indirect means of assessing the effect of the applied force. Specifically, by comparing how ductal network properties (ductal length, branching angles etc) in both the TENSION (left, experimental) and CONTRA (right, experimental) glands change relative to right CTL glands, across conditions in which uniaxial force was applied for 2 weeks, applied for 1 week, or applied for 1 week and then released for 1 week, we assessed the consistency of the observed effects across experiments. This approach is analogous to using a fixed reference point to evaluate relative variation across independent experiments, and provides an indirect way to contextualize the CONTRA observations in the absence of left CTL controls. Analysis of the CONTRA glands relative to CTL was performed post hoc, motivated by our observation of increased ductal length and altered branching angles in the TENSION glands. Because the CONTRA analysis was not part of the original study design, corresponding left CTL glands were not collected. In hindsight, inclusion of these samples for a direct comparison of left CONTRA to left CTL glands would have provided an additional control and strengthened the analysis.

We acknowledge that mounting, fixing, and staining procedures can introduce variations. Consistent application of these procedures across all experimental and control groups means that any preparation-induced variation would affect groups equally, and thus cannot account for directional differences observed between conditions. Furthermore, the reproducibility of findings across the primary and secondary experiments, conducted at different times and processed by different individuals, supports this interpretation, though we acknowledge this does not eliminate the possibility of preparation-induced variation entirely.

This study captured morphological development in the x and y planes only. It is possible that application of tension through gluing also affected gland thickness and ductal growth in the z-direction, which could not be assessed using the current whole mount preparation and imaging approach. Future studies employing volumetric imaging methods will be needed to fully characterize the three-dimensional effects of mechanical force on mammary gland morphogenesis. While external application of the uniaxial tension enabled analysis of changes in mechanical force during in vivo mammary gland pubertal ductal development, the gluing and need for reapplication of glue likely resulted in variability in force application and may have contributed to the variability in the metrics captured. In both experiments, glue remained intact for the first 3–4 days following application, after which mice more actively removed it, requiring increasingly frequent reapplication. Despite this limitation, the repeatability of findings across both experiments, particularly with respect to changes in branching angles, supports the conclusion that externally applied tension affects mammary gland branching morphogenesis. The glue or mouse removal of the glue also caused irritation, and potentially inflammation, in the skin overlying the mammary glands, which could affect ductal development [54]. Notably, CONTRA glands, which had no direct application of glue nor any immediately associated irritation or inflammation, also responded to the uniaxial tension, substantiating the interpretation that application of uniaxial tension modifies mammary ductal branch orientation. Ongoing studies are aimed at elucidating mechanisms of these changes in ductal branching, which may potentially include remodeling of ECM, inflammatory mediators, and cellular signaling.

There are a couple of possible explanations for changes in length in primary experiments, despite no changes in width, curvature, or area. One may be changing the shape of the fat pad in the z-direction, which cannot be accurately characterized in 2D microscopy images. Another possible explanation is that force changes the arrangement of the stroma, which influences the branching angle. We know from Hannezo et al. [51] that a random walk model that is only biased in bifurcation angle is enough to reveal the emergence of a global bias along the proximal-distal orientation. It is likely this global bias is exaggerated due to local bias in smaller branching angles due to force. We can infer that an increase in smaller branching angles, as shown in Fig 6A and 6B, would increase the length along the proximal-distal direction. As we have shown, a locally biased random walk model is enough to increase the length of a ductal network(Fig 7). A global and small local bias would increase the overall length, while not increasing the width. The area does not change since the total area of the fat pad does not grow or shrink due to force.

This idea is consistent in the secondary experimental results as well. The release of tension early in development may be rearranging the stroma, leading to larger branching angles. The larger branching angles would increase ductal formation along the positive and negative sides of the gland, rather than the proximal-distal direction. The area would also be smaller since the area measures the span of the ductal network. Thus, the area of the ductal network cannot be larger than the area of the fat pad, but it may be smaller. In the secondary experiments, the ductal network in TENSION spanned a smaller gland area than CTL in the 7-weeks old mice (Fig 8). This implies that the ductal network spanned a smaller region of the gland than CTL.

In summary, findings from this study indicate that mechanical forces may regulate the orientation of ductal branches and the overall length of the ductal network. Improving our understanding of the mammary ductal network formation is important because the epithelial ductal network affects the subsequent development of milk-secreting glandular tissue, and thus may impact long-term milk production. An improved understanding of how mechanics impacts the formation of the epithelial ductal network is essential for understanding factors that may lead to developmental abnormalities. It may also yield insights into the formation of other branched organs and may potentially lead to the identification of novel ways to design artificial organs to combat diseases [55].

## Supporting information

S1 Fig A practice mouse that was not used in this experiment was utilized to demonstrate how the mouse nipples were sealed down. A) demonstrates the area surrounding the nipple after being plucked to remove the hair, B) shows how the skin was rolled to cover the nipple after the topical adhesive was applied, and C) shows how the skin appears after the adhesive dries.
(TIF)

S2 Fig Demonstration of skeletonized segmentation procedure A) shows a raw mammary gland microscopy image, B) shows the segmentation after passing through a neural network, and C) shows the result after errors were corrected manually in Adobe Photoshop. Manual correction was used to remove noisy pixels and reconnect any disconnected branches.
(TIF)

S3 Fig Demonstration of morphometrics A-B) Shows the process for straightening the skeletal outlines of the microscopy images. A curved line is used to trace the midline through the long axis of the gland. This line is straightened in ImageJ using the straighten tool such that the curve is a horizontal line. C) Demonstrates the curve that is entered in the Kappa plugin to determine the curvature of the ductal network.
(TIF)

S4 Fig Shows the process for identifying the nipple in images where it is not visible. A) shows how the outflow of branches is traced back to a source and B) shows how that source line marks the nipple for length/width measurements.
(TIF)

S5 Fig Shows the various length (red) and width (blue) measurements taken as a boundary box that starts from the key location as the left boundary and the distal tip as the right boundary. A) shows the bounding box from the nipple to the distal tip, B) shows the bounding box from the beginning of the lymph node to the distal tip. C) shows the bounding box from the end of the lymph node to the distal tip. The black bar is 1 mm.
(TIF)

S6 Fig Additional morphometrics for primary experiment. Demonstrates that A-B) curvature of glands is slightly greater in CONTRA versus CTL glands, but there is no difference in the number of perimetral endbuds. Violin plots, with median indicated by horizontal line. * indicates significance at p < 0.1. In (A), the y-axis is truncated to emphasize differences within the observed range.
(TIF)

S7 Fig Additional morphometrics for secondary experiment. Shows morphometric significance with outliers removed. A-E) show width morphometrics for mice in the secondary experiment at 6 weeks of age, and F-J) show width morphometrics for mice in the secondary experiment at 7 weeks of age.
(TIF)

S1 Text Supplementary Information of the paper. (PDF)
