# Supplementary figures and images for: Application of a uniaxial force by pulling the skin around the mammary gland may affect the orientation of the ducts and the length of the mammary ductal network: Findings from computational modeling and laboratory experiments
Source: PLoS Comput Biol. 2026 Jul 14;22(7):e1014421. doi: 10.1371/journal.pcbi.1014421 (PMC13384402; doi:10.1371/journal.pcbi.1014421)

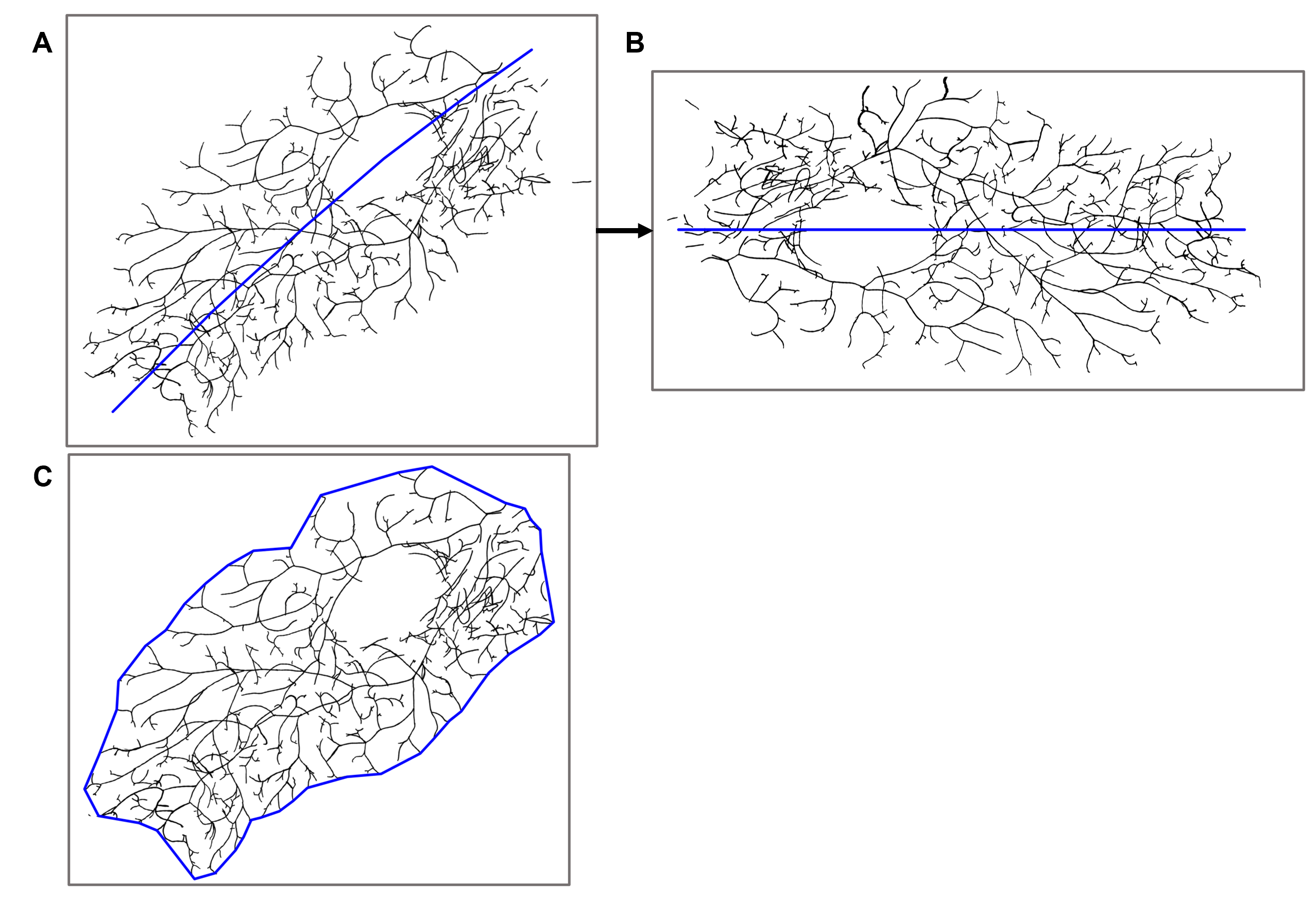

Supplement: S3 Fig — A curved line is used to trace the midline through the long axis of the gland. This line is straightened in ImageJ using the straighten tool such that the curve is a horizontal line. C) Demonstrates the curve that is entered in the Kappa plugin to determine the curvature of the ductal network. (TIF) [file pcbi.1014421.s003.tif]

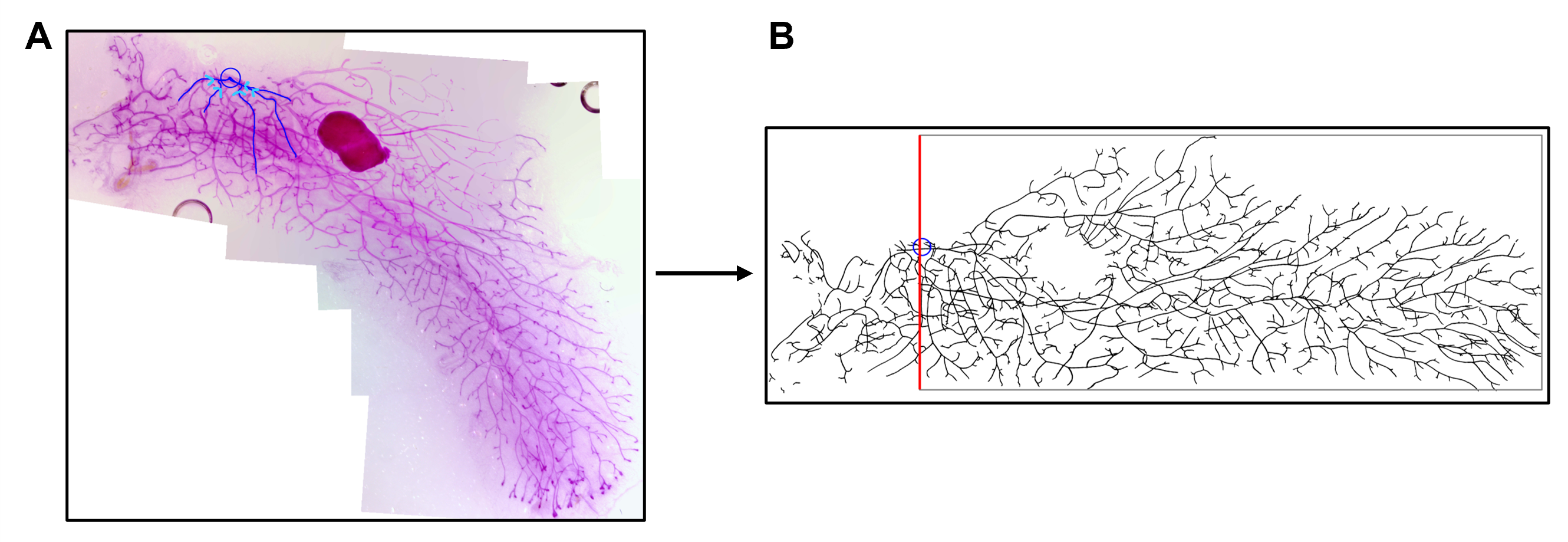

Supplement: S4 Fig — A) shows how the outflow of branches is traced back to a source and B) shows how that source line marks the nipple for length/width measurements. (TIF) [file pcbi.1014421.s004.tif]
