# Supplementary material for: Application of a uniaxial force by pulling the skin around the mammary gland may affect the orientation of the ducts and the length of the mammary ductal network: Findings from computational modeling and laboratory experiments
Source: PLoS Comput Biol. 2026 Jul 14;22(7):e1014421. doi: 10.1371/journal.pcbi.1014421 (PMC13384402; doi:10.1371/journal.pcbi.1014421)
Supplement: S1 Text — (PDF) [file pcbi.1014421.s008.pdf]

Supplementary Materials

Application of a uniaxial force by gluing the skin around the mammary gland may affect the length and branching angles of the mammary ductal network: Findings from computational modeling and laboratory experiments

Daisy Ulloa, Kelsey M. Teeple, Sara B. Scinto, Wonders O. Ogundare, Deloris D. Franklin, Theresa M. Casey, Uduak Z. George*

*Corresponding author (ugeorge@sdsu.edu)

The PDF file includes:

Supplementary Text

Tables A to F

Application of force by gluing the skin surrounding the nipple

Using images of actual mice, we demonstrate in Fig S1 how force was applied by gluing the skin surrounding the nipple.

Morphometric illustrations

This section illustrates how morphometric measures were captured. The skeletonized segmentation images are captured by passing a raw micrscopy images into a neural network, then hand-correcting areas that are over- and under-filled (S2 Fig). To straighten the glands, a curved line following the midline of the mammary glands is drawn. The image is straightened about this line in ImageJ (S3A and S3B Fig). The curvature is captured by tracing the perimeter of the glands in the Kappa plugin (S3C Fig). The process for identifying the nipple for length measurements can be seen in S4 Fig. Three length measurements were taken: nipple to distal tip (S5A Fig), start of the lymph node to distal tip (S5B Fig), and end of the lymph node to distal tip (S5C Fig).

Additional morphometric information

In this section, the additional figures for morphometric measures mentioned in the main text are shown. In S6 Fig, curvature and perimetral endbud counts for the primary experiment are shown. Width, curvature, and perimetral endbud counts for the secondary experiment for mice aged 6 weeks and 7 weeks are in S7 Fig.

We have also included the p-value adjustments when outliers are removed in Table A From these results, we can see curvature in the primary experiment tends to be larger in CONTRA glands compared to CTL ($p_* = 0.07$). This curvature may be due to the one-sided pulling force CONTRA glands were exposed to, as seen in Fig 1C. This can also be seen in the secondary experimental CONTRA glands as well, for mice at 6 weeks.

Experiment	Morphometric	Significance direction	p-value with outliers	p-value after removal of outliers
Primary	Curvature	CONTRA > CTL	0.30	0.065
Secondary (mice aged 6 weeks)	Curvature	TENSION < CTL	0.089	0.27
	Curvature	CONTRA > CTL	0.27	0.01

Table A. Shows all p-values that adjusted in significance level when outliers were removed for curvature morphometrics.

Unlike the primary experiments, we observed some slight differences in secondary 7-week experiments in the gland area spanned by the ductal network, width of the ductal network, and perimetral endbuds. The total gland area spanned by the ductal network in TENSION glands was significantly smaller than CTL ($p_* = 0.01$, Table 2). The width from the start of the lymph node to the distal tip between CONTRA and CTL also tended towards significance (p = 0.08), and the width from the end of the lymph node to the distal tip was significantly greater than CTL (p = 0.03, S7 Fig). Perimetral endbuds (i.e. those closest to the perimeter) are significantly increased CTL glands compared to TENSION (p = 0.01) and CONTRA glands (p = 0.01).

Comparisons of the angular positions of the ducts in TENSION and CONTRA to CTL for 5°, 10°, 20°, and 60° bin sizes

In Fig 5 A-C, we showed the distribution of the angular positions of the ducts using histograms with bins of 60° width. To further determine how the bin size affects the ability to detect the differences in

the angular positions of the ducts in TENSION and CONTRA compared to CTL, we analyzed the differences for bin size less than 90°. Tables B-D show the angular positions that were significantly different in TENSION and CONTRA compared to CTL for 5°, 10°, 20°, 30° bin sizes for primary and secondary experiments respectively. Columns 3-5 shows that we capture finer regions as we reduce the bin size from 60° to 5°. Using a bin size of 5° for the histogram allows us to identify finer regions that contribute more to the observed changes in ductal network orientation between TENSION versus CTL and CONTRA versus CTL. This finding is consistent with the prediction of the optimal bin size from the Wasserstein distance between the distributions for the angular positions as reported in the main text.

bin size	TENSION > CTL	CONTRA > CTL	TENSION < CTL	CONTRA < CTL
5		10° to 14° *<br>15° to 19° **<br>20° to 24° **<br>25° to 29° **<br>30° to 34° **	-90° to -86° *<br>50° to 54° *<br>60° to 64° *<br>65° to 69° *<br>85° to 89 *	-90° to -86° *<br>-75° to -71° *<br>-70° to -66° *<br>-65° to -61° *<br>-50° to -46° **<br>-45° to -41° ***<br>-40° to -36° *
10		10° to 19° **<br>20° to 29° **	60° to 69° *	-90° to -81° *<br>-80° to -71° **<br>-70° to -61° *<br>-50° to -51° **
20		10° to 29° **	50° to 69° **	-90° to -71° *<br>-70° to -51° *<br>-50° to -31° **
60		-30° to 29° *	30° to 89° *	-90° to -31° **

Table B. Primary experiment: Differences in the angular positions of the ductal network measured from the start of the lymph node to distal tip in TENSION and CONTRA compared to CTL. The angle ranges in the different columns are those that were significantly different in TENSION and CONTRA compared to CTL for different histogram bin sizes. Here, outliers were not removed. * $p < 0.1$, ** $p < 0.05$, *** $p < 0.01$.

In Table E and Table F, the changes in significance values for the primary and secondary experiments are recorded, respectively. Here, we noticeably see that TENSION tends towards smaller values compared to CTL for the primary experiments (Table E). We also see the opposite in secondary experiments, with TENSION tending towards a larger proportion of branching in larger angles compared to CTL.

bin size	TENSION > CTL	CONTRA > CTL	TENSION < CTL	CONTRA < CTL
5		-25° to -21° **<br>-20° to -16° ***<br>-15° to -11° *		45° to 49° **<br>50° to 54° **<br>55° to 59° *
10		-30 ° to -21° *<br>-20° to -11° **	-70° to -61° *	40° to 49° **<br>50 ° to 59° *
20		-30° to -11° **		30° to 49° *
60				

Table C. Secondary experiment for mice aged 6 weeks: Differences in the angular positions of the ductal network measured from the nipple to distal tip in TENSION and CONTRA compared to CTL. The angle ranges in the different columns are those that were significantly different in TENSION and CONTRA compared to CTL for different histogram bin sizes. Here, outliers were not removed. * $p < 0.1$, ** $p < 0.05$, *** $p < 0.01$.

bin size	TENSION > CTL	CONTRA > CTL	TENSION < CTL	CONTRA < CTL
5	-80° to -76° *<br>-75° to -71° **<br>-70° to -66° **<br>-65° to -61° **<br>-60° to -56° *<br>-25° to -21° *<br>60° to 64° *<br>65° to 69° **	45° to 49° *<br>50° to 54° **<br>55° to 59° **<br>60° to 64° **<br>65° to 69° *<br>70° to 74° **	0° to 4° **<br>5° to 9° **	-50° to -46° *<br>-40° to -36° *<br>-15° to -11° **<br>-10° to -6° **<br>-5° to -1° **<br>0° to 4° **
10	-80° to -71° *<br>-70° to -61° **<br>60° to 69° **	-80° to -71° *<br>50° to 59° **<br>60° to 69° **<br>70° to 79° **	0 ° to 9° **	-40 ° to -31° *<br>-20 ° to -11° *<br>-10 ° to -1° **
20		50° to 69° **<br>70° to 89° *	-10° to 9° *	-30 ° to -11 ° *<br>-10 ° to 9 ° **
60		30° to 89° ***		

Table D. Secondary experiment for mice aged 7 weeks: Differences in the angular positions of the ducts measured from the end of the lymph node to the distal tip in TENSION and CONTRA compared to CTL. The angle ranges in the different columns are those that were significantly different in TENSION and CONTRA compared to CTL for different histogram bin sizes. Here, outliers were not removed. * $p < 0.1$, ** $p < 0.05$, *** $p < 0.01$.

Experiment	Bin size	Significance direction	Angle	p-value	p* value
primary	5	TENSION > CTL	-15° to -11°	0.12	0.09
		CONTRA > CTL	-15° to -11 °	0.14	0.06
			10° to 14 °	0.07	0.12
			25° to 29°	0.03	0.06
		TENSION < CTL	-90° to -86°	0.1	0.03
			50° to 54°	0.08	0.03
			55° to 59°	0.12	0.05
		CONTRA < CTL	-90° to -86°	0.07	0.02
			-85° to -81°	0.19	0.08
			-80° to -76°	0.11	0.05
			-75° to -71°	0.09	0.25
			-40° to -36°	0.09	0.16
			85° to 89°	0.13	0.07
	10	TENSION > CTL	-20° to -11°	0.12	0.09
		TENSION < CTL	50° to 59°	0.12	0.05
		CONTRA < CTL	-90° to -81°	0.07	0.03
			-80° to -71°	0.05	0.0006
			80° to 89°	0.17	0.08
	20	TENSION < CTL	50° to 69°	0.05	0.002
		CONTRA < CTL	-90° to -71°	0.06	0.02
			-50° to -31°	0.05	0.08
			50° to 69°	0.33	0.07
	60	TENSION > CTL	-90° to -31°	0.21	0.05
		TENSION < CTL	30° to 89°	0.07	0.007
		CONTRA < CTL	-90° to -31°	0.02	0.004
			30° to 89°	0.3	0.06

Table E. Shows all p-values computed after outliers are removed for the primary experiment. Only angles in Table B that changed in significance value are listed for the different bin sizes.

experiment	bin size	significance direction	angle	previous p-value	p* value
secondary (6wk)	5	TENSION > CTL	-55° to -51°	0.2	0.09
			15° to 19°	0.3	0.07
		CONTRA > CTL	-55° to -51 °	0.21	0.03
			-25° to -21 °	0.02	0.0001
			-15° to -11 °	0.06	0.01
		TENSION < CTL	-90° to -86°	0.14	0.008
			45° to 49°	0.17	0.09
			55° to 59°	0.14	0.02
			85° to 89°	0.1	0.08
	10	CONTRA > CTL	-30° to -21°	0.09	0.007
			-20° to -11°	0.03	0.006
		TENSION < CTL	-90° to -81°	0.17	0.03
			50° to 59°	0.21	0.04
			80° to 89°	0.1	0.06
		CONTRA < CTL	-80° to -81 °	0.19	0.1
			50° to 59 °	0.08	0.02
	20	CONTRA > CTL	-30° to -11 °	0.01	0.002
		TENSION < CTL	-90° to -71°	0.12	0.05
secondary (7wk)	5	TENSION > CTL	-80° to -76°	0.1	0.15
		CONTRA > CTL	85° to 89 °	0.2	0.09
		TENSION < CTL	0° to 4 °	0.03	0.06
			20° to 24 °	0.17	0.08
		CONTRA < CTL	-50° to -46 °	0.06	0.03
			-40° to -36 °	0.08	0.19
			-30° to -26 °	0.1	0.05
			-15° to -11 °	0.03	0.06
	10	TENSION > CTL	-80° to -71°	0.07	0.12
		TENSION < CTL	0° to 9°	0.04	0.06
			20° to 29°	0.12	0.03
			40° to 49°	0.11	0.05
		CONTRA < CTL	-20° to -11°	0.08	0.21
	20	TENSION > CTL	-90° to -71°	0.14	0.07
		CONTRA > CTL	10° to 29°	0.17	0.06
		TENSION < CTL	-10° to 9°	0.1	0.16
			30° to 39°	0.17	0.07
	60	CONTRA < CTL	-90° to -31°	0.2	0.03

Table F. Shows all p-values computed after outliers are removed for the secondary experiments. Only angles in Table C and Table D that changed in significance value are listed for the different bin sizes.
